# Transmission and pathogenicity in ferrets after experimental infection with HPAI clade 2.3.4.4b H5N1 viruses

**DOI:** 10.1099/jgv.0.002124

**Published:** 2025-07-08

**Authors:** Luca Bordes, Nora M. Gerhards, Marit Roose, Sandra Venema, Marc Engelsma, Wim H.M. van der Poel, Evelien A. Germeraad, Nancy Beerens, Sandra Vreman

**Affiliations:** 1 Wageningen Bioveterinary Research, Lelystad, Netherlands

**Keywords:** avian influenza, ferret, H5N1, PB2-E627K, transmission

## Abstract

A marked increase in the incidence of mortality amongst wild mammals attributed to infection with highly pathogenic avian influenza (HPAI) H5N1 clade 2.3.4.4b viruses was observed in Europe in 2021. Neurological signs and high viral antigen levels in the brain of infected wild mammals indicate that the HPAI H5N1 virus causes severe disease in mammals, but serological analysis suggests that infections may be more widespread, implying that some mammals could experience mild disease. The clinical manifestation and transmissibility of HPAI H5N1 clade 2.3.4.4b viruses amongst mammals represent critical risk factors for potential zoonotic transmission to humans. This study examined the pathogenicity, viral tissue tropism and associated pathology of three HPAI H5N1 viruses in ferrets, which are a model for influenza A infection in humans. Ferrets were experimentally infected with an HPAI H5N1 poultry virus (genotype C) and two HPAI H5N1 viruses (genotype BA) isolated from a red fox, one of which carries the zoonotic PB2-E627K mutation. The red fox isolate, but not the poultry isolate, caused high morbidity, viral shedding and mortality in ferrets. Transmission to co-housed ferrets was investigated in a group setting for the virus carrying the PB2-E627K mutation and caused neurological signs accompanied by prominent viral antigen staining in recipient ferrets compared to directly inoculated ferrets. This study shows that HPAI H5N1 clade 2.3.4.4b viruses can infect mammals with varying pathogenicity and that mammal-to-mammal transmission can occur. This increases the zoonotic potential of the virus and highlights the need for enhanced surveillance in wild mammals for early detection of potential zoonotic threats.

## Data availability

All sequence data is publicly available via GenBank.

A/chicken/Netherlands/20019879-001005/2020: PV888726: PV888733

A/red fox/Netherlands/21040099-006_PB2E/2021: PV888736: PV888743

A/red fox/Netherlands/21040099-006_PB2K/2021: PV888745: PV888752

## Introduction

Highly pathogenic avian influenza (HPAI) H5N1 viruses were introduced in Europe in 2020, causing large-scale epizootics in poultry, wild birds and occasionally infections in mammalian species [1]. Transatlantic spread in December 2021 caused subsequent large-scale epizootics in North America and South America [2], and outbreaks in poultry continued, also during the summer periods until 2023 on all these continents [3]. The HPAI H5N1 clade 2.3.4.4b genotype C virus emerged in late 2020 but did not cause a large epizootic and was quickly followed up by other genotypes (e.g. AB, AC and BA) in 2021 which resulted in the largest epizootic recorded in Europe to date [4]. Introductions into mammalian species occurred in wild carnivorous mammals such as red foxes (*Vulpes vulpes*), polecats (*Mustela putorius*), otters (*Lutra lutra*) and badgers (*Meles meles*), but also in marine mammals such as harbour seal (*Phoca vitulina*), grey seal (*Halichoerus grypus*) and harbour porpoise (*Phocoena phocoena*) [5,15]. Genetic analysis of the viruses isolated from infected wild carnivorous land and sea mammals suggested that the virus was introduced by consumption of infected bird carcasses, but horizontal transmission to close contacts could not be ruled out [7,16, 17]. Mammal-to-mammal transmission may have occurred amongst sea lions along the coasts of Peru and Chile; however, the outbreak subsided, suggesting that further viral adaptations are necessary for sustained transmission between mammalian hosts [18]. Neurological signs were frequently observed in infected wild carnivorous mammals corroborated by high amounts of viral antigen in the brain [7,17]. The severity and extension of the infection in the respiratory tissues were ambiguous. Next to case studies in diseased wild mammals, serological analyses indicated that HPAI infections may be more widespread in carnivorous mammals than indicated by the recorded mortalities, implying that some mammals could experience a milder form of infection without showing obvious clinical signs [19]. Occasionally, HPAI H5N1 infections were detected in companion animals such as domestic cats and dogs in France, Poland, Italy and North America [20,23]. HPAI H5N1 was also detected in farmed minks in Spain [24]. Furthermore, in March 2024, an outbreak of the North American HPAI H5N1 clade 2.3.4.4b virus occurred in ruminants and continued to infect numerous dairy herds in multiple states [25]. Experimental infection through the udder of cattle and infection of well-differentiated bovine airway epithelial cells with European HPAI H5N1 clade 2.3.4.4b viruses indicated that multiple lineages of HPAI H5N1 may have the propensity to infect cattle [25,28]. Infections of mammalian livestock and domestic animals pose a more significant risk for human transmission than wild carnivorous mammals, considering their close interaction with humans. Although multiple wild, domestic and livestock mammals were infected with HPAI H5N1 clade 2.3.4.4b, only 15 human cases were reported to the World Health Organization between 2022 and 2024, of which four individuals had severe clinical signs [27]. Since 2024, the number of human HPAI H5N1 cases has surged in the USA, with 70 reported infections, including one fatality [29].

Experimental infection with recent HPAI H5N1 clade 2.3.4.4b viruses in ferrets demonstrated varying clinical signs ranging from mild disease and no transmission between co-housed ferrets [30] to severe disease and transmission to co-housed ferrets [31,33]. This indicates that pathogenicity and transmissibility under experimental conditions in ferrets are variable for the currently circulating H5N1 viruses and that the severity of the infection and transmission are likely dependent on specific genetic viral traits. Mammalian adaptations including PB2-E627K and PB2-D701N have been detected in multiple H5N1 viruses isolated from mammals and are known to increase pathogenicity by adaptation to the lower temperatures of the mammalian respiratory tract compared to avian species [6,34, 35]. However, some viruses isolated from infected wild mammals contain no known mammalian adaptations, indicating that the currently circulating HPAI H5N1 clade 2.3.4.4b viruses contain unknown adaptive mutations, underlining the need for further studies.

Ferrets are a well-established model for studying influenza A virus infections in humans which closely resemble the severe clinical signs observed in humans infected with HPAI and the transmissibility of seasonal influenza viruses [36,37]. The resemblance of ferrets to humans in lung physiology, cellular receptor distribution and the manifestation of clinical signs positions them as a valuable model for studying influenza virus infection and transmission dynamics and assessing pandemic risks in human populations [38]. In this study, ferrets were experimentally infected with an HPAI H5N1 poultry virus (genotype C) and two HPAI H5N1 viruses (genotype BA) isolated from a red fox, one of which carries the zoonotic PB2-E627K mutation. Additionally, the direct transmission of the virus carrying the zoonotic mutation PB2-E627K was studied in a group setting.

## Methods

### Virus preparation sequencing and phylogenetic analysis

The following three virus strains were used in this study: (1) A/chicken/Netherlands/20019879-001005/2020 (H5N1-2020-C), EPI_ISL_17791407, originating from an index case on a poultry farm in the Netherlands; (2) A/red fox/Netherlands/21040099-006_PB2E/2021 (H5N1-2021 PB2-627E); and (3) A/red fox/Netherlands/21040099-006_PB2K/2021 (H5N1-2021 PB2-627K), respectively EPI_ISL_19057416 and EPI_ISL_19057417, originating from the same red fox brain homogenate detected in the Netherlands. The fox isolates were separated by serial dilution in 9- to 11-day-old specific pathogen-free (SPF) embryonated chicken eggs (ECEs, obtained from Royal GD, Deventer, the Netherlands), as described previously [6]. Viruses were passaged twice in 9- to 11-day-old SPF ECE (further referred to as E2 passage). Whole-genome sequencing was performed both on the seed material and E2 passage as described previously [39]. An in-depth analysis of the sequencing data did not reveal any minority virus populations (threshold 1%) or nucleotide changes between the original virus isolate and E2 passages. One additional mutation (G485R) in the nucleoprotein (NP), which was already present in the original isolate, remained in the E2 passage of the H5N1-2021 PB2-627E virus isolate. End-point titrations on 9- to 11-day-old SPF ECEs were performed to determine the median egg infective dose (EID_50_) of the virus isolates. The virus isolates were titrated in triplicate on different days, and the EID_50_ titres were calculated using the method of Reed and Muench [40]. Virus stocks were diluted in 2.95% tryptose phosphate buffer (TPB) to 10^6^ EID_50_ per millilitre for inoculation.

For each genome segment, phylogenetic analysis of the complete genome sequences was performed, aligning the virus sequences using MAFFT v7.475 [41] and reconstructing the phylogeny using maximum likelihood (ML) analysis with IQ-TREE software v2.0.3 and 1,000 bootstrap replicates [42]. The R package ggtree was used to visualize the ML tree [43]. The submitters of the GISAID sequences used in the phylogenetic analysis are acknowledged in Table S1, available in the online Supplementary Material. Host shift adaptations were identified using the FluMutGUI version 3.1.1 and FluMutDB 6.2 (released on 15 July 2024) [44].

### Experimental design

**Fig. 1.**
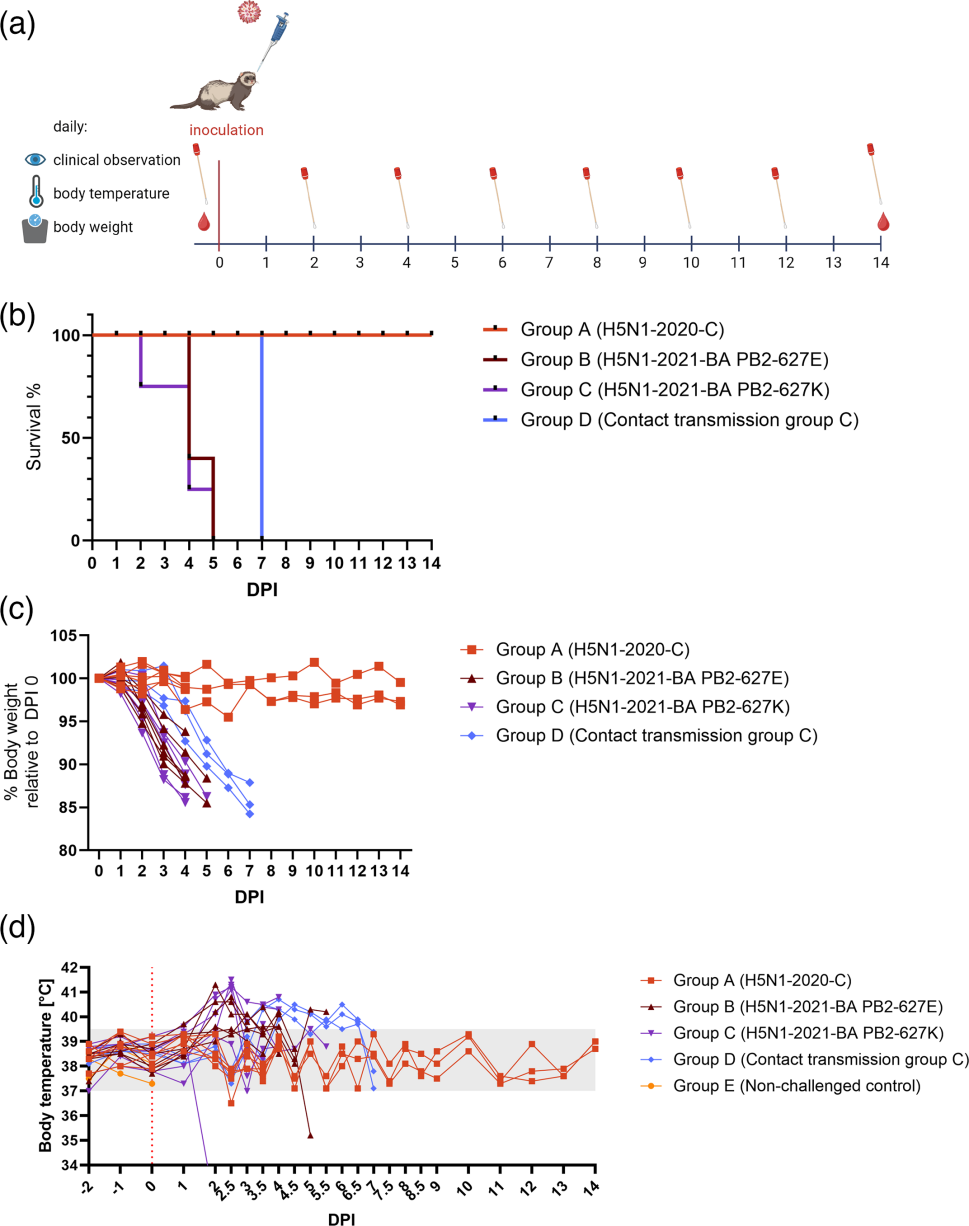
Survival and clinical parameters. (**a**) Schematic outline of the study design. (**b**) Survival post-inoculation showing only ferrets that reached the HEP or were found dead (*n*=1); animals scheduled for necropsies on 0 DPI (group E) and on 4 DPI (groups A, **B and **C) are not shown. (**c**) Relative body weight compared to the day of inoculation. (**d**) Body temperature measured by a subcutaneous transponder. The shaded area indicates the body temperature considered within the physiological range which was measured before the challenge inoculation (37–39.5 °C). Hypothermic animals were humanely euthanized on 2 and 5 DPI. Group E was euthanized on 0 DPI.

For this study, *N*=24 castrated and descented male ferrets were obtained from Triple F Farms LLC, USA. They were vaccinated against distemper and rabies and were 35 weeks of age upon arrival. The animals were randomized on body weight and housed in one stable divided into three pens of equal size (4.5 m^2^). During the 14-day acclimatization period, *N*=7 animals were housed in the first pen, *N*=8 animals in the second pen and *N*=9 animals in the third pen. Sawdust was used as bedding material. As cage enrichment, hammocks, shelters, tunnels and towels were provided. Commercial ferret feed and fresh drinking water were provided *ad libitum*. On the day of inoculation, *N*=1 ferret from the first pen and *N*=2 ferrets from the second pen were euthanized as control animals. Ferrets in the first pen (group A) were inoculated with H5N1-2020 (Table 1, Fig. 1a). Ferrets in the second pen (group B) were inoculated with H5N1-2021 PB2-627E. From the third pen, *N*=3 animals attributed to group D were spatially separated from the *N*=6 animals attributed to group C prior to inoculation with H5N1-2021 PB2-627K. After 8 h, the spatial separation between groups C and D was removed, allowing direct contact between these groups. Inoculations were performed by pipetting 0.1 ml of inoculum drop-wise into each nostril, resulting in a total dosage of 10^5.3^ EID_50_ per ferret. Inoculations, as well as blood and swab sampling, were performed under general anaesthesia using 0.1 mg kg^−1^ medetomidine and 5 mg kg^−1^ ketamine, injected intramuscularly in the hind limb, which was antagonized by injecting 0.1 mg kg^−1^ atipamezole intramuscularly in the other hind limb. All anaesthetics were obtained from AST Farma, Oudewater, the Netherlands. Serum blood samples (2.5 ml) were collected once on −7 days post-inoculation (DPI) to confirm seronegativity for influenza A virus. On this day, a subcutaneous temperature transponder (Thermochip Mini, Sure Petcare) was injected to facilitate non-invasive temperature measurements. Body temperature was measured once a day between −6 and 1 DPI as well as between 9 and 14 DPI, and twice a day between 2 and 8 DPI. Body weights were measured every other day during the acclimatization period, and daily from −1 DPI until the end of the study. Swabs were collected every other day from 0 DPI onwards from the nose, oropharynx and anus. Additionally, swabs were collected from ferrets that reached a humane endpoint (HEP). Clinical signs were evaluated once on −1 and 0 DPI, and twice daily post-inoculation using a specific scoring scheme encompassing scores from 0 (absent) to 3 (severe) clinical signs. The following parameters were evaluated: depression, nasal discharge, sneezing/coughing, breathing, skin changes, neurological signs and diarrhoea (details scoring Table S2). HEPs were applied when the following criteria were met: a combined score of 4 for depression, breathing (dyspnoea) and neurological signs (e.g. tremors); a score of 3 for either breathing or neurological signs; a score of 2 for breathing (e.g. moderately increased respiratory rate or abdominal breathing) persisting for over 48 h; or a weight loss exceeding 20% of the animal’s body weight compared to the day of inoculation (not observed in this experiment). All animals that reached the HEP were euthanized and necropsied immediately.

**Table 1.** Experimental groups and source of the viruses

Group	Virus	Source	Infection route	No. of animals
A	H5N1-2020	Poultry	Intranasal inoculation	6
B	H5N1-2021 PB2-627E	Red fox	Intranasal inoculation	6
C	H5N1-2021 PB2-627K	Red fox	Intranasal inoculation	6
D	H5N1-2021 PB2-627K	Transmission	Direct transmission	3
E	No virus	na	na	3

na, not applicable.

### Pathology and immunohistochemistry

Euthanasia was performed by terminal blood collection through the vena cava caudalis and the aorta under general anaesthesia, as described above. All ferrets were evaluated for macroscopic changes, and the lungs were weighed before further processing. Of each animal, conchae, trachea, lung, brain (cerebrum, cerebellum and olfactory bulb), trigeminal ganglion, tonsil, heart, liver, spleen, kidney, pancreas, duodenum, ileum and colon were collected in 10% neutral buffered formalin and snap-frozen in liquid nitrogen. The left lung lobes were inflated with formalin, processed routinely and embedded in paraffin. Immunohistochemistry (IHC) for the detection of the presence of influenza A NP and haematoxylin and eosin (HE) stain for the evaluation of histopathological changes were performed as described previously [40]. Histopathology and IHC were assessed by a board-certified veterinary pathologist in an unblinded fashion.

The IHC stain was semi-quantitatively evaluated for the extension of viral expression and was graded: no staining (grade 0), focal or multifocal, <5 foci, sparse staining (grade 1), moderate staining, multifocal, >5 foci (grade 2), abundant staining, multifocal to coalescing (grade 3) and excessive staining, almost diffuse staining [4]. The HE stain was semi-quantitatively evaluated for characteristics and severity of histopathologic changes and was scored subjectively on a scale of 0–3 with 0 indicating no influenza virus-associated lesion and 3 indicating severe influenza virus-related lesions.

### Viral RNA and antibody detection

Swabs (oropharyngeal, nasal and anal) were placed in 2 ml of 2.95% TPB and frozen at −70 °C before processing. Collected tissues were snap-frozen in liquid nitrogen and stored at −70 °C before processing. The frozen tissues (lung, liver, olfactory bulb and cerebrum) of approximately equal size (5 mm^2^) were homogenized in 1.0 ml TPB with the MagNaLyser (Roche, Basel, Switzerland). Standard curves were generated by making tenfold dilutions of the virus stocks in 2.95% TPB and subsequently freezing the log10 −2 to −9 dilutions at −70 °C. Avian influenza virus (AIV) RNA from swab material, tissue material and standard curves was extracted using the MagNA Pure 96 system (Roche, Basel, Switzerland). AIV RNA was detected by a quantitative real-time RT-PCR targeting the matrix gene (M-PCR), as described previously [39]. The standard curves were used to calculate EID_50_ equivalents. Oropharyngeal swabs and tissue homogenate (lung, liver, olfactory bulb and cerebrum) with a cycle threshold (Ct) below 36 were additionally titrated on MDCK cells to quantify infectious virus particles as described above. Detection of antibodies against AIV NP was performed by using an in-house NP-ELISA as described previously [45]. Swabs and serum collected during the acclimatization period were tested directly, without freezing, for the presence of AIV RNA by M-PCR and AIV antibodies by NP-ELISA. Swabs and serum collected after inoculation of the ferrets were tested after freezing at −70 °C and −20 °C, respectively.

### Minority virus population sequencing

All oropharyngeal swabs with a Ct below 33 were analysed for minority virus populations by whole-genome sequencing as described above. The emergence of the PB2-E627K mutation was further investigated in a selection of homogenized tissues from group B (H5N1-2021 PB2-627E) by a PCR targeting the PB2-E627K mutation followed by sequencing on Illumina’s MiSeq as described previously [6].

## Results

### Genetic analysis and potential host shift adaptations

All three HPAI H5N1 viruses belonged to H5 clade 2.3.4.4b and were highly related to wild bird and mammalian viruses isolated during the subsequent epizootics. The largest genetic distance between the H5N1-2020 and two fox viruses was identified on PB2 (97.06% nucleotide similarity), and minor genetic distances were identified on the remaining six segments (98.88%–99.22% nucleotide similarity) (Fig. S1). Virus sequences were screened for previously identified virulence factors known to influence virulence, host specificity or binding of host proteins using FluMut [44]. Besides the PB2-E627K mutation, two additional mutations, PB2-A588V [46,47] and NP-I41V [46,48], were identified (not on an H5 background) on both fox isolates which may be involved in increased polymerase activity.

### Survival and clinical parameters

During acclimatization and likely due to stress associated with transport, two ferrets lost 17% of their body weight until the day of inoculation (results not shown). One of these animals was euthanized on 0 DPI as an uninfected control animal, whilst the other one (ferret #21) was inoculated with H5N1-2021 PB2-627K (group C). All ferrets from group A survived until the end of the study (14 DPI) and showed no clinical signs throughout the experiment (Fig. 1b). All inoculated ferrets from groups B and C showed clear clinical signs. From the animals which were scheduled for necropsy on 4 DPI, the HEP was not reached before the scheduled necropsy time, except for ferret #21, which reached HEP on 2 DPI (Table S3). Animals which were scheduled for necropsy but did not reach the HEP before the necropsy date were excluded from the survival analysis.

All inoculated ferrets from group B or C which were scheduled for necropsy on 14 DPI reached the HEP between 4 and 5 DPI. All three contact transmission ferrets (group D) reached the HEP on 7 DPI. Inoculated ferrets in groups B and C showed substantial body weight loss (15% body weight loss compared to 0 DPI) and developed body temperatures ≥39.5 °C from 1 DPI onwards (Fig. 1c, d). Similar findings were observed in transmission group D with the onset of an increase in body temperature on 3 DPI. The most frequently observed clinical signs were depression and ataxia (mild neurological signs), next to respiratory distress, which was observed in almost all animals in groups B, C and D (Table S3). Notably, only animals in group D exhibited moderate to severe neurological signs (grade 2 or 3), with one animal displaying pronounced neurological tremors. No clinical signs were observed in group A.

### Virus shedding and antibody development

**Fig. 2.**
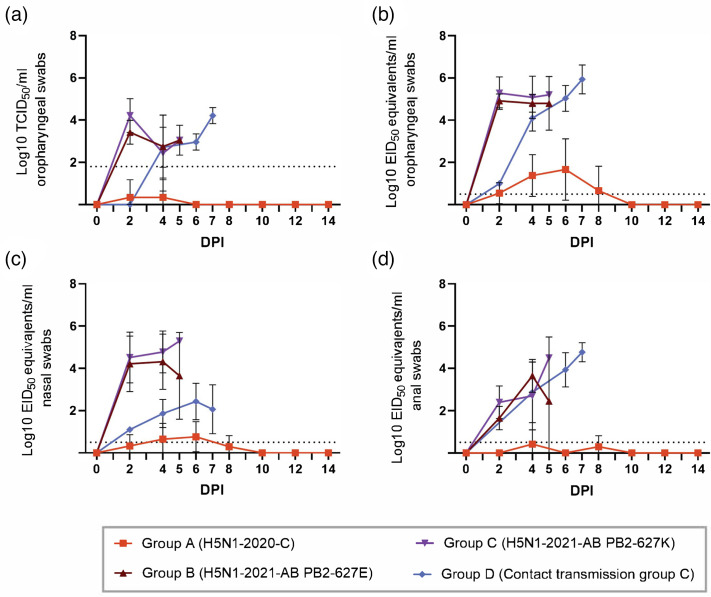
Virus shedding in swabs. (**a**) Viral litres in oropharyngeal swabs with a Ct <36 were determined using endpoint titration. Swabs with a Ct ≥36 were assigned an infectious virus titre of 0. The dotted line indicates the detection limit of the test (≤10^1.8^ TCID_50_ per millilitre). (**b**) Viral loads in oropharyngeal swabs. (**c**) Viral loads in nasal swabs. (**d**) Viral loads in anal swabs. (**b**, **c **and d) were determined by quantitative reverse transcription PCR (qRT-PCR) and calculated as EID_50_ equivalents per millilitre. The dotted line indicates the detection limit of the test (≤10^0.5^ TCID_50_ per millilitre).

Oropharyngeal swabs from groups B and C collected on 2 DPI to 5 DPI contained high amounts of viral RNA (~10^5^ EID_50_ equivalents per millilitre) (Fig. 2b). Oropharyngeal swabs were further titrated on MDCK cells to quantify infectious virus particles. Shedding of infectious virus particles closely resembled viral RNA shedding patterns, although the infectious virus litres were ~10² TCID_50_ per millilitre lower than the viral RNA loads (Fig. 2a). Slightly lower viral RNA loads (~10^4^ EID_50_ equivalents per millilitre) were measured in nose swabs collected from groups B and C, with a decreasing trend over time for group B which was not observed for group C (Fig. 2c). At 2 DPI, relatively low amounts of viral RNA were detected in anal swabs compared to oropharyngeal and nasal swabs, but viral RNA loads gradually increased for group C up to the HEP and for group B up to 4 DPI (Fig. 2d). The transmission group D exhibited an upward trend in viral shedding in oropharyngeal swabs, with viral RNA loads approaching 10^6^ EID_50_ equivalents per millilitre and infectious virus litres reaching up to 10^4^ TCID_50_ per millilitre (Fig. 2a, b). In contrast, viral RNA loads in nasal swabs from group D remained near the assay’s detection limit. Similar to groups B and C, the ferrets in group D showed an increase in virus shedding over time in the anal swabs. Virus shedding was minimal (near or below the detection limit) for all swab samples collected from group A, with the highest viral RNA loads in oropharyngeal swabs. Only two out of six oropharyngeal swabs from group A exceeded the detection threshold for infectious virus particles (10^2.05^ TCID_50_ per millilitre), resulting in an average infectious virus titre slightly above 0 on 4 and 6 DPI.

Before the start of the study (−7 DPI), all ferrets were negative for influenza-specific antibodies. Only the three ferrets of group A, which were euthanized on 14 DPI, developed influenza-specific antibodies, whilst no influenza-specific antibodies were detected in any of the other ferrets after infection (data not shown).

### HPAI-related pathology and virus expression in tissues

The uninfected control ferrets (*n*=3) and the ferrets from group A (*n*=6) showed no macroscopic changes upon the scheduled necropsies on 0, 4 and 14 DPI. The main macroscopic findings of ferrets from groups B, C and D were pale mottled livers with foci of necrosis and oedema and necrosis in the pancreas (Fig. S2), which were observed in all ferrets except for ferret #10 (group B). There was no substantial increase in relative lung weight compared to the body weight in any of the ferrets, which was corroborated by minimal macroscopic changes in the lung and trachea, except for ferret #20 (group C, found dead in stable) and ferret #21 (group C, which had already shown severe respiratory distress at 2 dpi) (Table S3).

**Fig. 3.**
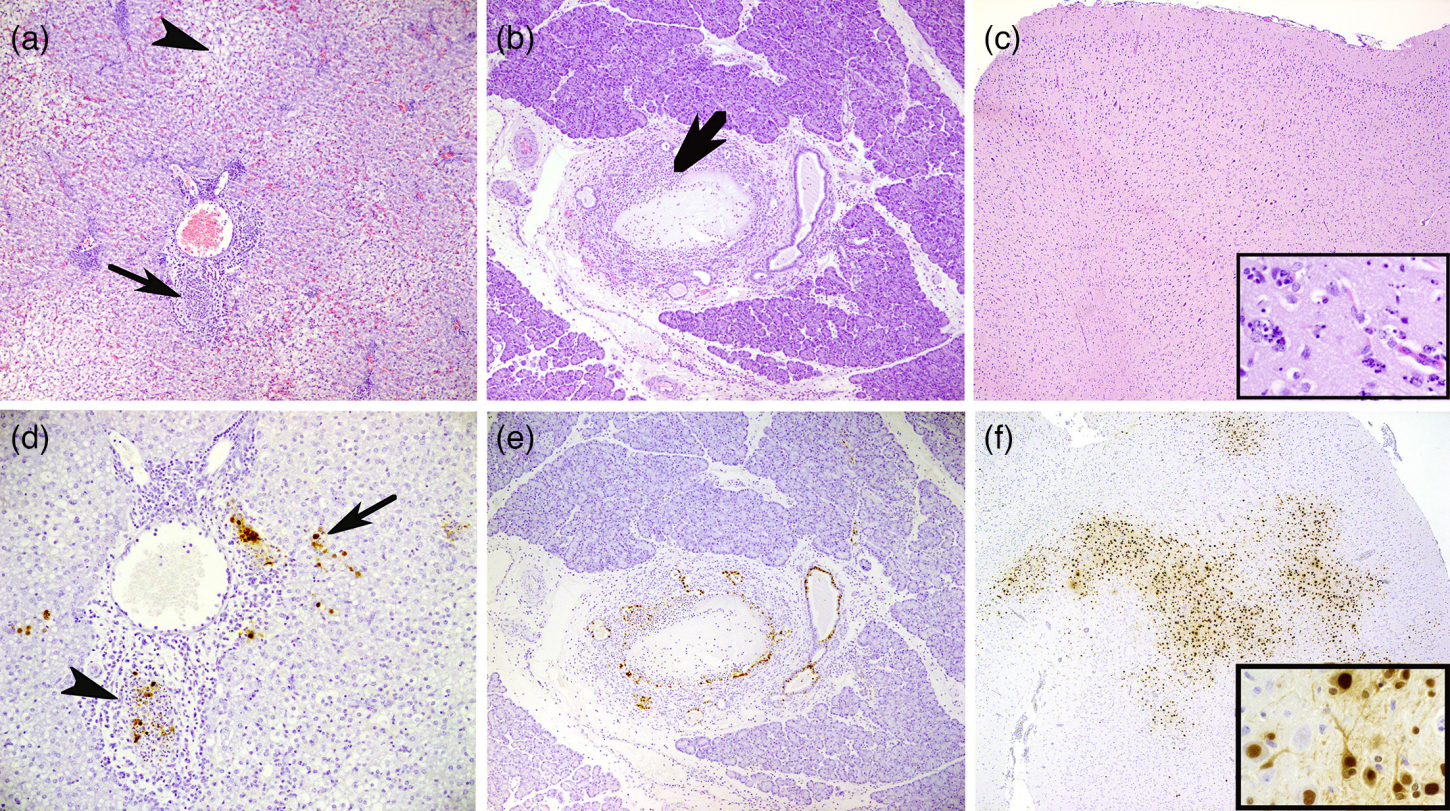
H5N1-induced pathology and associated virus expression. (**a**) Liver, vacuolization of hepatocytes (arrowhead), bile duct necrosis (arrow), ferret group C, objective 10×; (**b**) pancreas, necrosis in large duct (arrow), ferret group B, objective 10×; (**c**) brain (cerebrum), gliosis and encephalomalacia, ferret group C, objective 5× and 40× (inset); (**d**) liver, moderate NP expression (grade 2) in sloughed epithelial cells of bile duct (arrowhead) and hepatocytes, Kupffer cells (arrow), ferret group B, objective 20×; (**e**) pancreas, moderate NP expression (grade 2) in epithelial cells of pancreatic ducts, ferret group C, objective 20×; (**f**) brain (cerebrum), extensive NP expression (grade 3) in glia cells and neurons, ferret group C, objective 5× and 40× (inset); (a–c) HE stain; (d–f) IHC influenza A NP corresponding with HE.

The most severe histopathologic lesions, consistent with the macroscopic changes, were observed in the pancreas and the liver (grade 3 score HE), with similar morphologic changes in groups B, C and D. Ferrets from group D showed dispersed necrosis with haemorrhage and hepatocyte vacuolization in the liver. Interestingly, there was also prominent necrosis of epithelial cells in bile ducts. This necrosis of ductal epithelial cells was also observed in the pancreas (Fig. 3a, b). The changes in the intestine were mild to moderate (Fig. S3). In all ferrets from groups B, C and D, there was moderate rhinitis with necrosis in olfactory and respiratory epithelial cells. The histopathological changes in the central nervous system (CNS) were mild and restricted to the trigeminal ganglion (Fig. S3), olfactory bulb and cerebrum. Group D showed more severe changes in the cerebrum compared to groups B and C, characterized by an increase in glia cells and necrosis of neurons/glia cells (encephalomalacia) (Fig. 3c). No clear histopathological changes were observed in the cerebellum and the thoracic spinal cord in all ferrets. Control ferret #13 showed moderate liver lipidosis without other significant findings. The other uninfected control ferrets and ferrets of group A showed no significant histopathological changes in the investigated tissues.

**Fig. 4.**
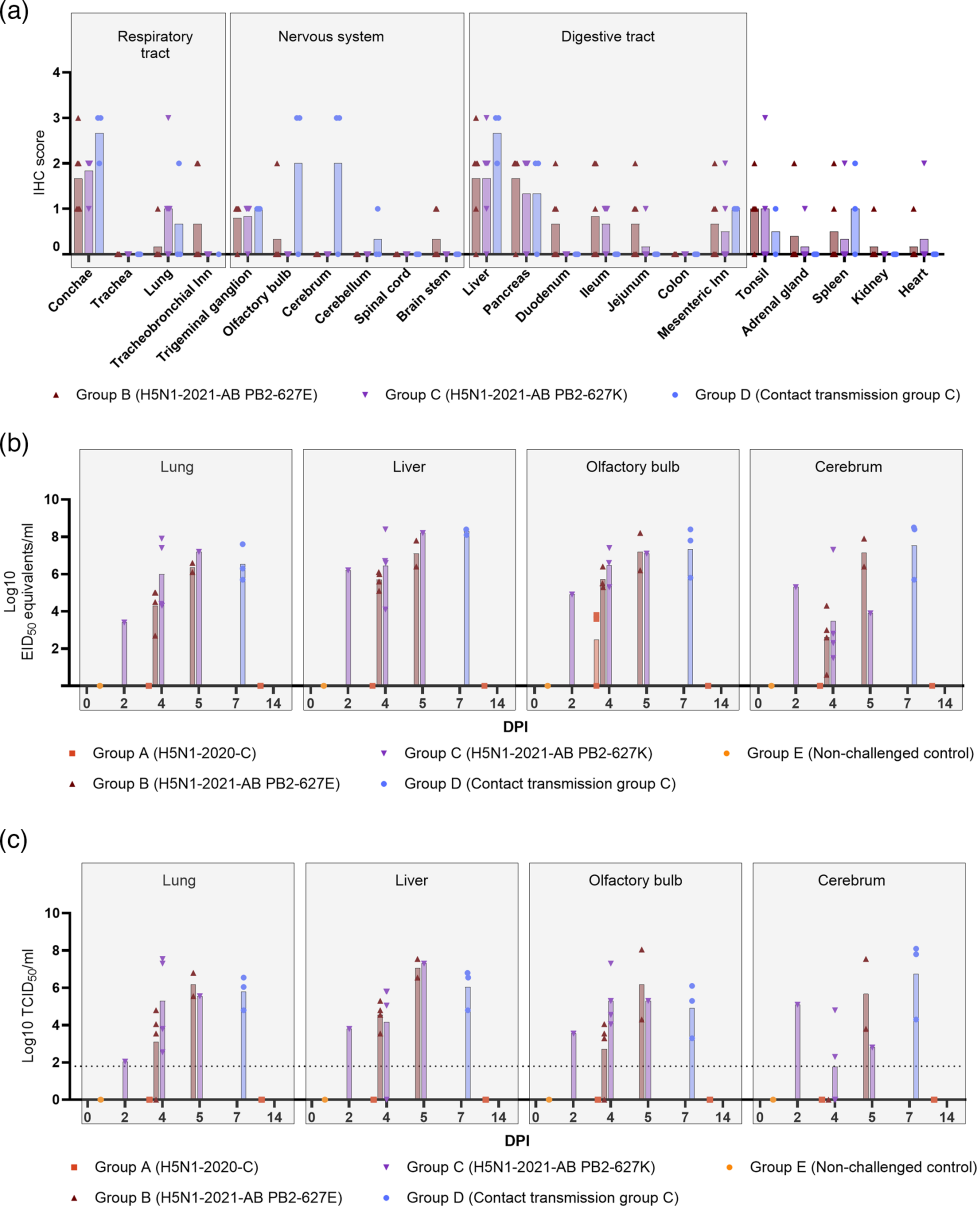
Viral protein and viral RNA in tissues. (**a**) Semiquantitative IHC scores of influenza A NP staining in various tissues of ferrets from groups B, C or D. Symbols depict individual score, and the bar represents the mean score. Ferrets from groups A and E were not included, no positive staining. (**b**) Viral loads in selected organ samples, as determined by qRT-PCR and expressed as EID_50_ equivalents per millilitre. The day of necropsy is indicated on the x-axis. Note that *N*=3 animals from group E were euthanized on DPI 0 and *N*=3 animals from group A on DPI 4 and on DPI 14 for comparison to the other groups, whilst ferrets from groups B (*n*=3) and C (*n*=2) had a scheduled necropsy on 4 DPI or were euthanized due to reaching an HEP or died spontaneously (groups B, C and D). **(c**) Infectious virus litres determined by endpoint titration of samples that had a Ct value <36 by qRT-PCR. Samples with Ct ≥36 were not tested but were assigned a titre 0. The dotted line indicates the detection limit of the test (≤10^1.8^ TCID_50_ per millilitre).

IHC was applied to investigate viral antigen expression in tissues and associated histopathology. Uninfected control ferrets and ferrets from group A showed no viral antigen in any of the investigated tissues. For groups B, C and D, viral antigen expression was most prominent in nasal conchae, liver and pancreas (mostly grade 2), with individual variation between the animals and no clear differences between groups or necropsy days (Fig. 4a). In the pancreas and liver, viral antigen was mainly found in ductal epithelial cells, but also in mononuclear cells (Fig. 3d, e). Also, in the nasal conchae, there was a prominent epithelial expression (both olfactory and respiratory epithelium) with variation between ferrets. There was minimal to moderate antigen expression in the lungs of individual animals dispersed throughout the groups, except for ferret #20, which showed extensive staining (grade 3) in the lungs (Fig. S3). Within the CNS, there was mild viral antigen expression in the trigeminal ganglion (grade 1) of almost all ferrets of groups B, C and D, whilst only two out of three ferrets of group D showed clear antigen expression in the olfactory bulb and cerebrum (grade 3). Viral antigen was expressed in neurons and microglia cells (Fig. 3f). The intestinal tract showed less viral expression compared to liver, pancreas and nasal conchae. Within individual animals dispersed throughout the groups, there was mild to moderate viral antigen staining in mononuclear cells of the ileum, but also in the neuronal plexus of the duodenum (Fig. S3). An overview of viral antigen expression in all investigated tissues is provided in Fig. 4a.

Additionally, we evaluated the viral RNA levels in the lung, liver and CNS (cerebrum and olfactory bulb) of groups A–D. In group A, minimal viral RNA was detected in the olfactory bulb at 4 DPI in two out of three ferrets, with no viral RNA identified in any other tissues. At 14 DPI, no viral RNA was detected in any of the examined organs of the ferrets in group A. This is in contrast to the lung, liver and CNS of all ferrets of groups B, C and D, in which comparable viral RNA levels were measured (Fig. 4b). Organs that had a Ct value <36 were subjected to endpoint titration (Fig. 4c), and for most samples, a similar pattern but with lower infectious litres was measured compared to viral RNA levels. Notably, the two olfactory bulb samples from group A that were positive by qRT-PCR tested negative by virus titration.

### Full genome sequencing and screening for PB2-E627K

Oropharyngeal swabs with sufficient viral loads (Ct <33) were selected for whole-genome sequencing to detect adaptations to the mammalian host. In total, 23 swabs were analysed [group B (*n*=7), group C (*n*=7) and group D (*n*=9)]. All consensus sequences in the oropharyngeal swabs were identical to the inoculum, and no minority populations were identified (threshold 1%). Additionally, four organs (lung, liver, olfactory bulb and cerebrum) were selected from the ferrets in group B (*n*=6) to identify the emergence of the PB2-E627K mutation in these tissues. One ferret (#11) showed a majority virus population of 87.61% with the PB2-E627K mutation in the liver and a minority virus population of 1.06% with the PB2-E627K mutation in the lung. No minority populations were identified in other organs or other ferrets of group B (results not shown). Finally, whole-genome sequencing was performed on cerebrum homogenates from ferrets in group D to determine whether the neurological signs observed in this group could be attributed to additional mutations arising specifically in the brain. The viral genome sequences from the brain homogenates of ferrets 22 and 24 were identical to the group C inoculum (donor ferrets), whereas two mutations (NP-G346A and NS-G386A) were identified in the cerebrum homogenate of ferret 23.

## Discussion

This study investigated the pathogenicity, tissue tropism and related pathology of three European HPAI H5N1 clade 2.3.4.4b viruses by experimental infection in ferrets. The H5N1-2020 poultry isolate did not induce clinical signs, shedding of infectious virus or infection-associated pathological changes. In contrast, both mammalian H5N1 isolates, regardless of the presence or absence of the PB2-627K mutation, caused severe clinical manifestations, shedding of infectious virus and corresponding pathological alterations. These were accompanied by significant expression of viral antigens (as detected by IHC) in the liver, pancreas and nasal conchae, as well as within the CNS of contact transmission animals.

Ferrets inoculated with the mammalian H5N1 isolates shed high levels of viral RNA in oropharyngeal and nasal swabs, whilst slightly lower viral RNA loads were measured in the anal swabs. In contrast, ferrets inoculated with the poultry isolate (group A) shed low levels of viral RNA in all swabs, but no infectious virus particles could be detected in the oropharyngeal swabs of this group. However, infectious virus was detected in the oropharyngeal swabs of groups B, C and D, indicating that the RNA-positive samples in group A represent remnants of the inoculum. Organ homogenates from ferrets inoculated with the poultry isolate (group A) were all negative for viral RNA, with the exception of the olfactory bulb which was positive by qRT-PCR but negative by virus titration. Therefore, the H5N1-2020 virus may cause an abortive infection in the olfactory bulb. Influenza A-specific antibodies could be detected at 14 DPI in these ferrets, which indicates that minor virus replication has likely occurred in the upper respiratory tract and/or olfactory bulb in these ferrets. Thus, not all HPAI H5N1 clade 2.3.4.4b viruses from the 2020–2024 epizootic may have the propensity to infect mammals and cause severe disease. This may explain the relatively limited number of detected mortalities in wild mammals compared to the number of serologically positive wild mammals [7,19]. Wild mammals are likely infected by a diverse range of HPAI H5N1 genotypes which may result in varying clinical outcomes ranging from severe disease, characterized by mortality and neurological signs, to asymptomatic infections without abundant viral shedding. Other genotypes may contain further adaptations to mammals causing asymptomatic infections with high viral shedding.

A surprisingly small difference in pathogenicity was observed between ferrets infected with the H5N1-2021 PB2-627E or 627K viruses, given that the PB2-E627K mutation is generally associated with enhanced viral polymerase activity in mammalian cells [6,34, 35] and can result in more severe disease in mammals [49,50]. The absence of enhanced pathogenicity associated with the PB2-E627K mutation has been previously observed in mice and ferrets, likely due to a compensatory mutation at positions 590 out of 591 (not present in these H5N1 viruses) [51,52]. This could suggest that either an unidentified compensatory mutation is responsible for the high pathogenicity of the HPAI H5N1-2021 PB2-627E virus in ferrets or that the H5N1-2021 PB2-627E virus rapidly mutated into the PB2-627K variant. The latter possibility was investigated by whole-genome sequencing of oropharyngeal swabs and screening for the PB2-627K variant in lung, liver, olfactory bulb and cerebral tissues. Only a single liver sample from ferrets inoculated with the H5N1-2021 PB2-627E virus exhibited a mutation to the PB2-627K variant, supporting the hypothesis that another mutation is responsible for the high morbidity and mortality observed in group B. Phylogenetic analysis of the PB2 segment revealed that the mammalian isolates contain a rare variant of the PB2 segment. This variant shows considerable divergence from the PB2 sequence of the poultry isolate and may have contributed to the severe clinical signs and extensive viral shedding observed in ferrets from group B.

Interestingly, ferrets infected with H5N1-2021 PB2-627K via direct contact transmission exhibited more pronounced neurological signs accompanied by expression of viral antigen in the olfactory bulb and cerebrum, compared to the ferrets which were directly inoculated. However, the viral RNA loads in brain samples of ferrets infected by direct transmission were comparable to those of inoculated ferrets. Furthermore, the two mutations NP-G346A and NS-G386A have not been previously associated with enhanced neurotropism, virulence, replication or transmission and were found in the brain of only one of the three ferrets in group D. One possible explanation for the less severe neurological signs in ferrets inoculated with the H5N1-2021 PB2-627K virus is the fact that they reached the HEP earlier after virus exposure (2–5 DPI) compared to ferrets infected by direct transmission, which were separated from the inoculated animals for only 8 h (HEP of contact animals at 7 DPI of the inoculated animals). Considering the histological lesions in the CNS, it is plausible that the lack of neurological signs in the inoculated ferrets is due to the rapid onset of mortality. Neurological signs may have manifested if the HEP had been reached 1–2 days later. Alternatively, it is equally possible that variations in viral dosage or the route of infection may have influenced the onset of neurological signs.

Efficient virus dissemination from the nasal epithelium to the CNS was observed, similar to other H5N1 viruses [53]. Neuronal viral protein staining was detected in the olfactory bulb, trigeminal ganglion, cerebrum, cerebellum and neurons of the duodenal plexus of Auerbach. This staining was prominent in numerous ferrets from groups B, C and D, further supporting the neurotropism of the studied viruses. Viral protein expression was also evident in the epithelial cells of bile and pancreatic ducts, which may have contributed to viral shedding through the intestine since minimal viral protein staining was observed in the intestinal mucosa. In most ferrets from groups B, C and D, severe disease leading to HEP was caused by a combination of moderate to severe necrotizing pancreatitis, hepatitis and encephalitis.

This study has several limitations. Although ferrets are a well-established model for studying influenza A virus infections in humans, results from the ferret model are not always directly translatable to human infections. During the acclimatization period, two ferrets lost a substantial fraction of their body weight. This was likely related to transport-induced stress. Even though these animals were offered additional feed, the body weight loss continued and resulted in hepatic lipidosis. One of the two animals could be euthanized as a control animal on 0 DPI, whilst the other one was challenged with H5N1-2021 PB2-627K. Besides the body weight loss, this animal was clinically healthy. However, post-challenge, this ferret quickly deteriorated, which resulted in euthanasia already on 2 DPI. Inoculation of ferrets with an additional wild bird isolate of the same genotype as the mammalian isolates could clarify whether the increased pathogenicity is a characteristic of the genotype or is dependent on specific genetic adaptations due to replication in mammals. Finally, direct transmission was evaluated in a group setting with physical contact, implying that individual infection timing is unknown and that virus shedding, pathology and sequencing results were shown on a group level. It would also have been interesting to assess the propensity for airborne transmission without contact and whether the PB2-627E virus is also able to efficiently transmit between individual ferrets.

Overall, this study shows that multiple HPAI H5N1 clade 2.3.4.4b genotypes have the propensity to infect mammals, but the pathogenicity can differ substantially between virus isolates. Furthermore, transmission between mammals was shown for one of the virus isolates, which underlines the zoonotic potential of current HPAI H5N1 viruses. Therefore, surveillance for HPAI H5 viruses should be intensified in wild mammals for early detection of zoonotic viruses.

## Supplementary material

10.1099/jgv.0.002124 Uncited Supplementary Material 1.

## Abbreviations

AIV: avian influenza virus
CNS: central nervous system
Ct: cycle threshold
DPI: days post-inoculation
ECE: embryonated chicken eggs
EID: egg infective dose
HE: haematoxylin and eosin
HEP: humane endpoint
HPAI: highly pathogenic avian influenza
IHC: immunohistochemistry
ML: maximum likelihood
NP: nucleoprotein
NP: nucleoprotein
qRT-PCR: quantitative reverse transcription PCR
SPF: specific pathogen-free
TPB: tryptose phosphate buffer
