# Supplementary material for: Transmission and pathogenicity in ferrets after experimental infection with HPAI clade 2.3.4.4b H5N1 viruses
Source: J Gen Virol. 2025 Jul 8;106(7):002124. doi: 10.1099/jgv.0.002124 (PMC12282323; doi:10.1099/jgv.0.002124)
Supplement: Uncited Supplementary Material 1. [file jgv-106-02124-s001.pdf]

## PB2

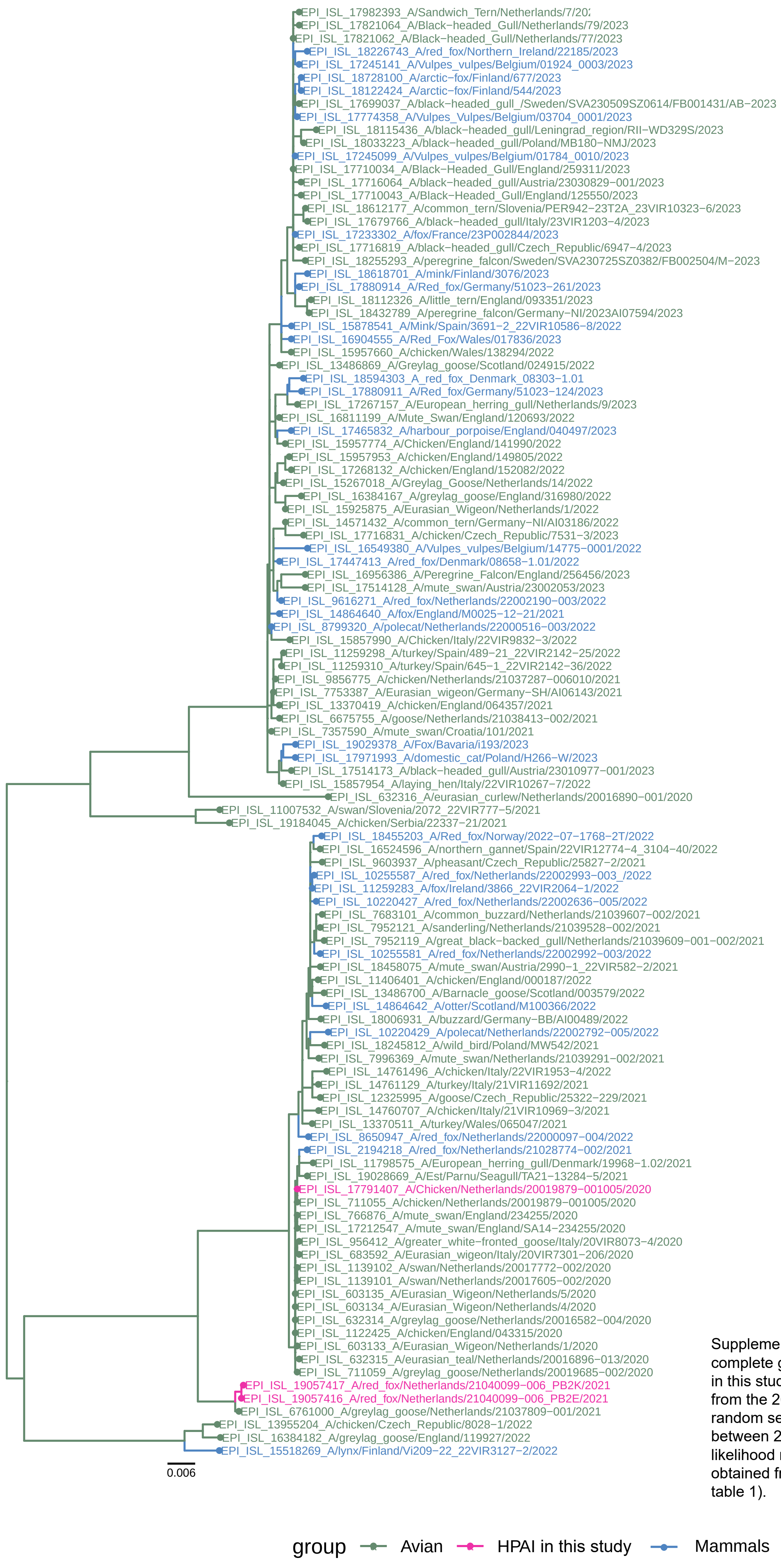


Supplementary Figure 1. Phylogenetic trees of the complete genome sequences with the strains used in this study (pink), a random set of bird isolates from the 2020-2023 epizootics (green) and a random set of mammalian isolates obtained between 2020-2023 (blue) inferred using maximum likelihood methods. Genome sequences were obtained from the GISAID database (Supplementary table 1).

PB1

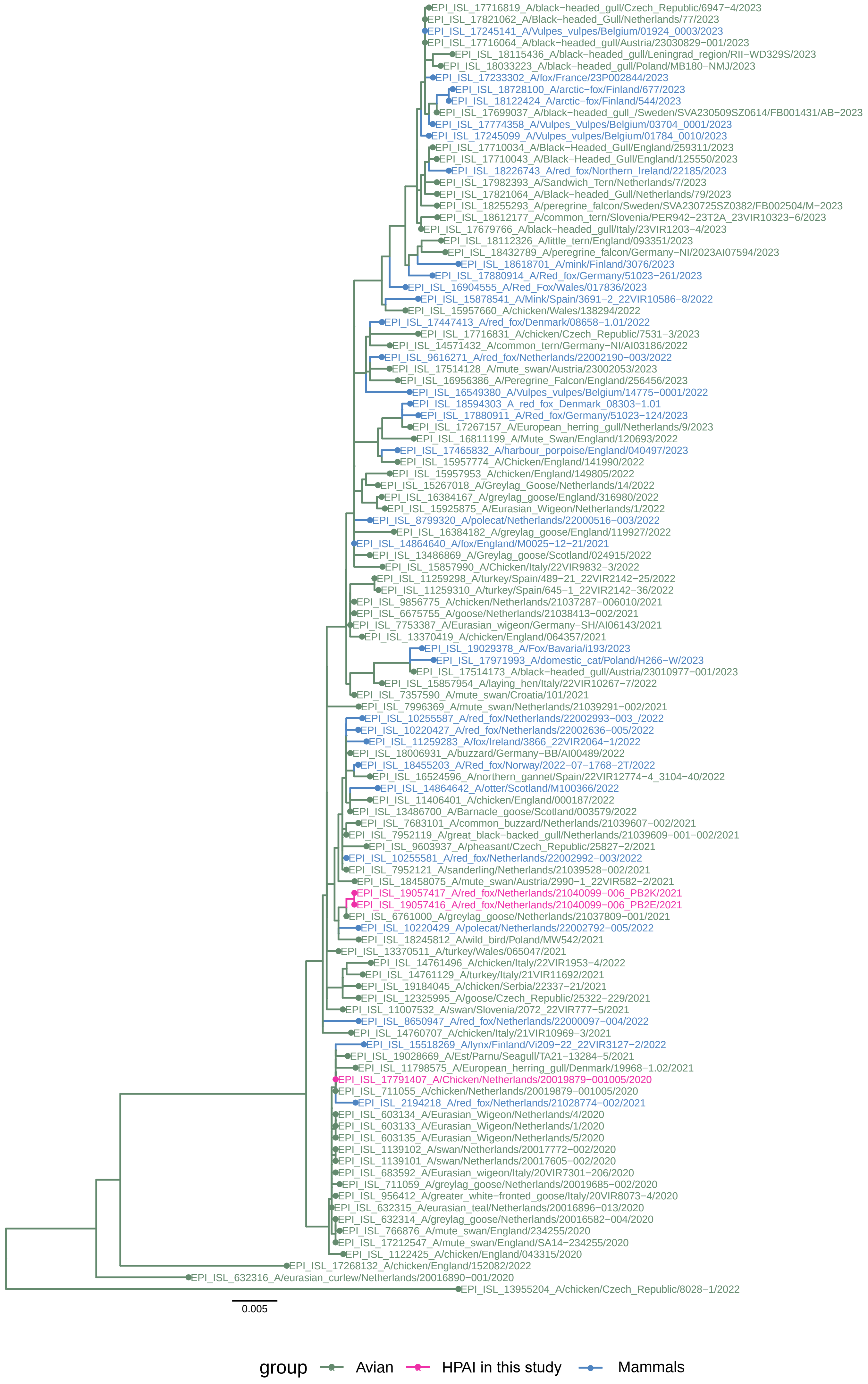

EPI_ISL_17716819_A/black−headed_gull/Czech_Republic/6947−4/2023
EPI_ISL_17821062_A/Black−headed_Gull/Netherlands/77/2023
EPI_ISL_17245141_A/Vulpes_vulpes/Belgium/01924_0003/2023
EPI_ISL_17716064_A/black−headed_gull/Austria/23030829−001/2023
EPI_ISL_18115436_A/black−headed_gull/Leningrad_region/RII−WD329S/2023
EPI_ISL_18033223_A/black−headed_gull/Poland/MB180−NMJ/2023
EPI_ISL_17233302_A/fox/France/23P002844/2023
EPI_ISL_18728100_A/arctic−fox/Finland/677/2023
EPI_ISL_18122424_A/arctic−fox/Finland/544/2023
EPI_ISL_17699037_A/black−headed_gull_/Sweden/SVA230509SZ0614/FB001431/AB−2023
EPI_ISL_17774358_A/Vulpes_Vulpes/Belgium/03704_0001/2023
EPI_ISL_17245099_A/Vulpes_vulpes/Belgium/01784_0010/2023
EPI_ISL_17710034_A/Black−Headed_Gull/England/259311/2023
EPI_ISL_17710043_A/Black−Headed_Gull/England/125550/2023
EPI_ISL_18226743_A/red_fox/Northern_Ireland/22185/2023
EPI_ISL_17982393_A/Sandwich_Tern/Netherlands/7/2023
EPI_ISL_17821064_A/Black−headed_Gull/Netherlands/79/2023
EPI_ISL_18255293_A/peregrine_falcon/Sweden/SVA230725SZ0382/FB002504/M−2023
EPI_ISL_18612177_A/common_tern/Slovenia/PER942−23T2A_23VIR10323−6/2023
EPI_ISL_17679766_A/black−headed_gull/Italy/23VIR1203−4/2023
EPI_ISL_18112326_A/little_tern/England/093351/2023
EPI_ISL_18432789_A/peregrine_falcon/Germany−NI/2023AI07594/2023
EPI_ISL_18618701_A/mink/Finland/3076/2023
EPI_ISL_17880914_A/Red_fox/Germany/51023−261/2023
EPI_ISL_16904555_A/Red_Fox/Wales/017836/2023
EPI_ISL_15878541_A/Mink/Spain/3691−2_22VIR10586−8/2022
EPI_ISL_15957660_A/chicken/Wales/138294/2022
EPI_ISL_17447413_A/red_fox/Denmark/08658−1.01/2022
EPI_ISL_17716831_A/chicken/Czech_Republic/7531−3/2023
EPI_ISL_14571432_A/common_tern/Germany−NI/AI03186/2022
EPI_ISL_9616271_A/red_fox/Netherlands/22002190−003/2022
EPI_ISL_17514128_A/mute_swan/Austria/23002053/2023
EPI_ISL_16956386_A/Peregrine_Falcon/England/256456/2023
EPI_ISL_16549380_A/Vulpes_vulpes/Belgium/14775−0001/2022
EPI_ISL_18594303_A_red_fox_Denmark_08303−1.01
EPI_ISL_17880911_A/Red_fox/Germany/51023−124/2023
EPI_ISL_17267157_A/European_herring_gull/Netherlands/9/2023
EPI_ISL_16811199_A/Mute_Swan/England/120693/2022
EPI_ISL_17465832_A/harbour_porpoise/England/040497/2023
EPI_ISL_15957774_A/Chicken/England/141990/2022
EPI_ISL_15957953_A/chicken/England/149805/2022
EPI_ISL_15267018_A/Greylag_Goose/Netherlands/14/2022
EPI_ISL_16384167_A/greylag_goose/England/316980/2022
EPI_ISL_15925875_A/Eurasian_Wigeon/Netherlands/1/2022
EPI_ISL_8799320_A/polecat/Netherlands/22000516−003/2022
EPI_ISL_16384182_A/greylag_goose/England/119927/2022
EPI_ISL_14864640_A/fox/England/M0025−12−21/2021
EPI_ISL_13486869_A/Greylag_goose/Scotland/024915/2022
EPI_ISL_15857990_A/Chicken/Italy/22VIR9832−3/2022
EPI_ISL_11259298_A/turkey/Spain/489−21_22VIR2142−25/2022
EPI_ISL_11259310_A/turkey/Spain/645−1_22VIR2142−36/2022
EPI_ISL_9856775_A/chicken/Netherlands/21037287−006010/2021
EPI_ISL_6675755_A/goose/Netherlands/21038413−002/2021
EPI_ISL_7753387_A/Eurasian_wigeon/Germany−SH/AI06143/2021
EPI_ISL_13370419_A/chicken/England/064357/2021
EPI_ISL_19029378_A/Fox/Bavaria/i193/2023
EPI_ISL_17971993_A/domestic_cat/Poland/H266−W/2023
EPI_ISL_17514173_A/black−headed_gull/Austria/23010977−001/2023
EPI_ISL_15857954_A/laying_hen/Italy/22VIR10267−7/2022
EPI_ISL_7357590_A/mute_swan/Croatia/101/2021
EPI_ISL_7996369_A/mute_swan/Netherlands/21039291−002/2021
EPI_ISL_10255587_A/red_fox/Netherlands/22002993−003_/2022
EPI_ISL_10220427_A/red_fox/Netherlands/22002636−005/2022
EPI_ISL_11259283_A/fox/Ireland/3866_22VIR2064−1/2022
EPI_ISL_18006931_A/buzzard/Germany−BB/AI00489/2022
EPI_ISL_18455203_A/Red_fox/Norway/2022−07−1768−2T/2022
EPI_ISL_16524596_A/northern_gannet/Spain/22VIR12774−4_3104−40/2022
EPI_ISL_14864642_A/otter/Scotland/M100366/2022
EPI_ISL_11406401_A/chicken/England/000187/2022
EPI_ISL_13486700_A/Barnacle_goose/Scotland/003579/2022
EPI_ISL_7683101_A/common_buzzard/Netherlands/21039607−002/2021
EPI_ISL_7952119_A/great_black−backed_gull/Netherlands/21039609−001−002/2021
EPI_ISL_9603937_A/pheasant/Czech_Republic/25827−2/2021
EPI_ISL_10255581_A/red_fox/Netherlands/22002992−003/2022
EPI_ISL_7952121_A/sanderling/Netherlands/21039528−002/2021
EPI_ISL_18458075_A/mute_swan/Austria/2990−1_22VIR582−2/2021
EPI_ISL_19057417_A/red_fox/Netherlands/21040099−006_PB2K/2021
EPI_ISL_19057416_A/red_fox/Netherlands/21040099−006_PB2E/2021
EPI_ISL_6761000_A/greylag_goose/Netherlands/21037809−001/2021
EPI_ISL_10220429_A/polecat/Netherlands/22002792−005/2022
EPI_ISL_18245812_A/wild_bird/Poland/MW542/2021
EPI_ISL_13370511_A/turkey/Wales/065047/2021
EPI_ISL_14761496_A/chicken/Italy/22VIR1953−4/2022
EPI_ISL_14761129_A/turkey/Italy/21VIR11692/2021
EPI_ISL_19184045_A/chicken/Serbia/22337−21/2021
EPI_ISL_12325995_A/goose/Czech_Republic/25322−229/2021
EPI_ISL_11007532_A/swan/Slovenia/2072_22VIR777−5/2021
EPI_ISL_8650947_A/red_fox/Netherlands/22000097−004/2022
EPI_ISL_14760707_A/chicken/Italy/21VIR10969−3/2021
EPI_ISL_15518269_A/lynx/Finland/Vi209−22_22VIR3127−2/2022
EPI_ISL_19028669_A/Est/Parnu/Seagull/TA21−13284−5/2021
EPI_ISL_11798575_A/European_herring_gull/Denmark/19968−1.02/2021
EPI_ISL_17791407_A/Chicken/Netherlands/20019879−001005/2020
EPI_ISL_711055_A/chicken/Netherlands/20019879−001005/2020
EPI_ISL_2194218_A/red_fox/Netherlands/21028774−002/2021
EPI_ISL_603134_A/Eurasian_Wigeon/Netherlands/4/2020
EPI_ISL_603133_A/Eurasian_Wigeon/Netherlands/1/2020
EPI_ISL_603135_A/Eurasian_Wigeon/Netherlands/5/2020
EPI_ISL_1139102_A/swan/Netherlands/20017772−002/2020
EPI_ISL_1139101_A/swan/Netherlands/20017605−002/2020
EPI_ISL_683592_A/Eurasian_wigeon/Italy/20VIR7301−206/2020
EPI_ISL_711059_A/greylag_goose/Netherlands/20019685−002/2020
EPI_ISL_956412_A/greater_white−fronted_goose/Italy/20VIR8073−4/2020
EPI_ISL_632315_A/eurasian_teal/Netherlands/20016896−013/2020
EPI_ISL_632314_A/greylag_goose/Netherlands/20016582−004/2020
EPI_ISL_766876_A/mute_swan/England/234255/2020
EPI_ISL_17212547_A/mute_swan/England/SA14−234255/2020
EPI_ISL_1122425_A/chicken/England/043315/2020
EPI_ISL_17268132_A/chicken/England/152082/2022
EPI_ISL_632316_A/eurasian_curlew/Netherlands/20016890−001/2020
EPI_ISL_13955204_A/chicken/Czech_Republic/8028−1/2022
0.005
group
Avian
HPAI in this study
Mammals

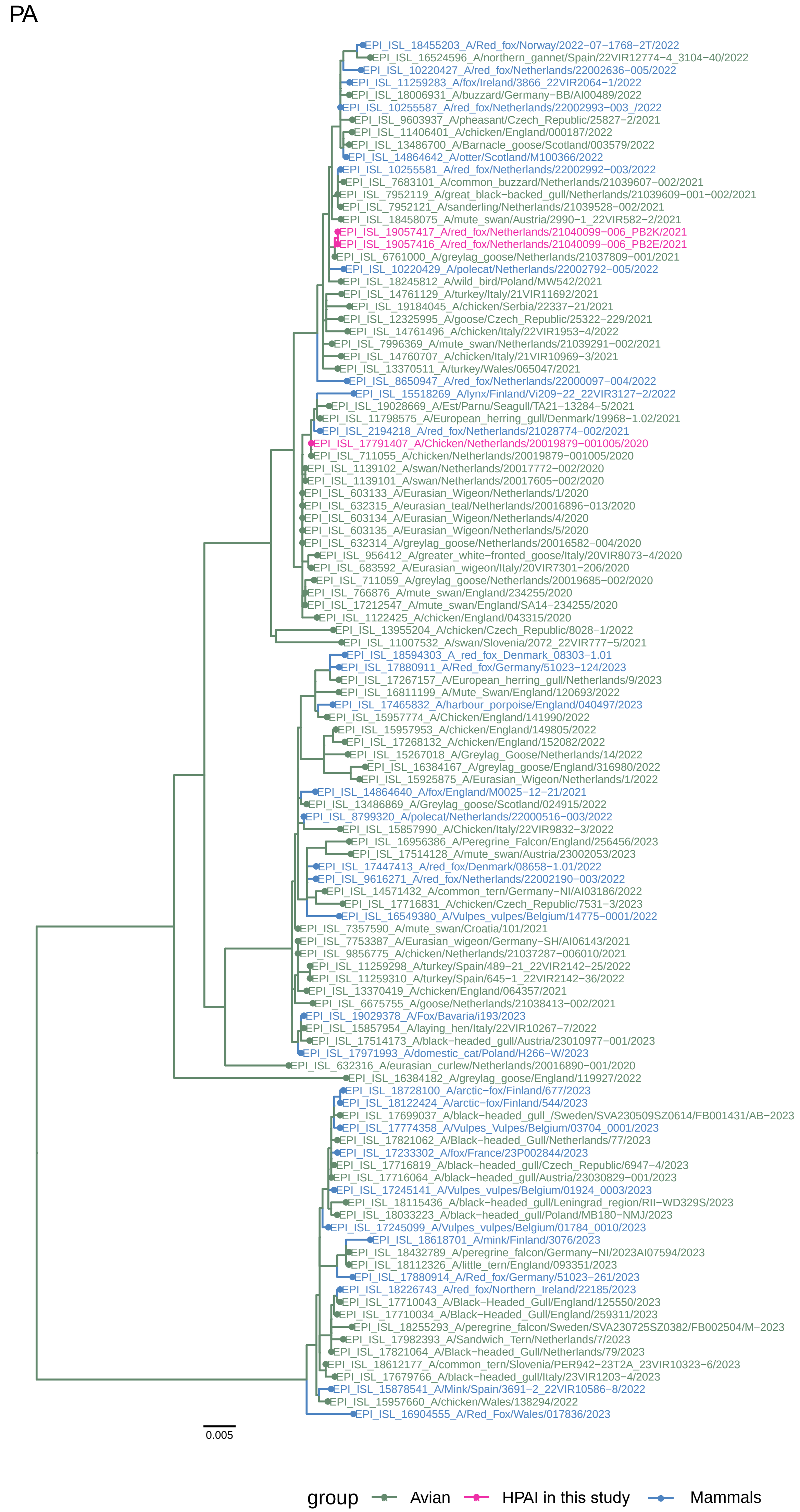
PA
EPI_ISL_18455203_A/Red_fox/Norway/2022−07−1768−2T/2022
EPI_ISL_16524596_A/northern_gannet/Spain/22VIR12774−4_3104−40/2022
EPI_ISL_10220427_A/red_fox/Netherlands/22002636−005/2022
EPI_ISL_11259283_A/fox/Ireland/3866_22VIR2064−1/2022
EPI_ISL_18006931_A/buzzard/Germany−BB/AI00489/2022
EPI_ISL_10255587_A/red_fox/Netherlands/22002993−003_/2022
EPI_ISL_9603937_A/pheasant/Czech_Republic/25827−2/2021
EPI_ISL_11406401_A/chicken/England/000187/2022
EPI_ISL_13486700_A/Barnacle_goose/Scotland/003579/2022
EPI_ISL_14864642_A/otter/Scotland/M100366/2022
EPI_ISL_10255581_A/red_fox/Netherlands/22002992−003/2022
EPI_ISL_7683101_A/common_buzzard/Netherlands/21039607−002/2021
EPI_ISL_7952119_A/great_black−backed_gull/Netherlands/21039609−001−002/2021
EPI_ISL_7952121_A/sanderling/Netherlands/21039528−002/2021
EPI_ISL_18458075_A/mute_swan/Austria/2990−1_22VIR582−2/2021
EPI_ISL_19057417_A/red_fox/Netherlands/21040099−006_PB2K/2021
EPI_ISL_19057416_A/red_fox/Netherlands/21040099−006_PB2E/2021
EPI_ISL_6761000_A/greylag_goose/Netherlands/21037809−001/2021
EPI_ISL_10220429_A/polecat/Netherlands/22002792−005/2022
EPI_ISL_18245812_A/wild_bird/Poland/MW542/2021
EPI_ISL_14761129_A/turkey/Italy/21VIR11692/2021
EPI_ISL_19184045_A/chicken/Serbia/22337−21/2021
EPI_ISL_12325995_A/goose/Czech_Republic/25322−229/2021
EPI_ISL_14761496_A/chicken/Italy/22VIR1953−4/2022
EPI_ISL_7996369_A/mute_swan/Netherlands/21039291−002/2021
EPI_ISL_14760707_A/chicken/Italy/21VIR10969−3/2021
EPI_ISL_13370511_A/turkey/Wales/065047/2021
EPI_ISL_8650947_A/red_fox/Netherlands/22000097−004/2022
EPI_ISL_15518269_A/lynx/Finland/Vi209−22_22VIR3127−2/2022
EPI_ISL_19028669_A/Est/Parnu/Seagull/TA21−13284−5/2021
EPI_ISL_11798575_A/European_herring_gull/Denmark/19968−1.02/2021
EPI_ISL_2194218_A/red_fox/Netherlands/21028774−002/2021
EPI_ISL_17791407_A/Chicken/Netherlands/20019879−001005/2020
EPI_ISL_711055_A/chicken/Netherlands/20019879−001005/2020
EPI_ISL_1139102_A/swan/Netherlands/20017772−002/2020
EPI_ISL_1139101_A/swan/Netherlands/20017605−002/2020
EPI_ISL_603133_A/Eurasian_Wigeon/Netherlands/1/2020
EPI_ISL_632315_A/eurasian_teal/Netherlands/20016896−013/2020
EPI_ISL_603134_A/Eurasian_Wigeon/Netherlands/4/2020
EPI_ISL_603135_A/Eurasian_Wigeon/Netherlands/5/2020
EPI_ISL_632314_A/greylag_goose/Netherlands/20016582−004/2020
EPI_ISL_956412_A/greater_white−fronted_goose/Italy/20VIR8073−4/2020
EPI_ISL_683592_A/Eurasian_wigeon/Italy/20VIR7301−206/2020
EPI_ISL_711059_A/greylag_goose/Netherlands/20019685−002/2020
EPI_ISL_766876_A/mute_swan/England/234255/2020
EPI_ISL_17212547_A/mute_swan/England/SA14−234255/2020
EPI_ISL_1122425_A/chicken/England/043315/2020
EPI_ISL_13955204_A/chicken/Czech_Republic/8028−1/2022
EPI_ISL_11007532_A/swan/Slovenia/2072_22VIR777−5/2021
EPI_ISL_18594303_A_red_fox_Denmark_08303−1.01
EPI_ISL_17880911_A/Red_fox/Germany/51023−124/2023
EPI_ISL_17267157_A/European_herring_gull/Netherlands/9/2023
EPI_ISL_16811199_A/Mute_Swan/England/120693/2022
EPI_ISL_17465832_A/harbour_porpoise/England/040497/2023
EPI_ISL_15957774_A/Chicken/England/141990/2022
EPI_ISL_15957953_A/chicken/England/149805/2022
EPI_ISL_17268132_A/chicken/England/152082/2022
EPI_ISL_15267018_A/Greylag_Goose/Netherlands/14/2022
EPI_ISL_16384167_A/greylag_goose/England/316980/2022
EPI_ISL_15925875_A/Eurasian_Wigeon/Netherlands/1/2022
EPI_ISL_14864640_A/fox/England/M0025−12−21/2021
EPI_ISL_13486869_A/Greylag_goose/Scotland/024915/2022
EPI_ISL_8799320_A/polecat/Netherlands/22000516−003/2022
EPI_ISL_15857990_A/Chicken/Italy/22VIR9832−3/2022
EPI_ISL_16956386_A/Peregrine_Falcon/England/256456/2023
EPI_ISL_17514128_A/mute_swan/Austria/23002053/2023
EPI_ISL_17447413_A/red_fox/Denmark/08658−1.01/2022
EPI_ISL_9616271_A/red_fox/Netherlands/22002190−003/2022
EPI_ISL_14571432_A/common_tern/Germany−NI/AI03186/2022
EPI_ISL_17716831_A/chicken/Czech_Republic/7531−3/2023
EPI_ISL_16549380_A/Vulpes_vulpes/Belgium/14775−0001/2022
EPI_ISL_7357590_A/mute_swan/Croatia/101/2021
EPI_ISL_7753387_A/Eurasian_wigeon/Germany−SH/AI06143/2021
EPI_ISL_9856775_A/chicken/Netherlands/21037287−006010/2021
EPI_ISL_11259298_A/turkey/Spain/489−21_22VIR2142−25/2022
EPI_ISL_11259310_A/turkey/Spain/645−1_22VIR2142−36/2022
EPI_ISL_13370419_A/chicken/England/064357/2021
EPI_ISL_6675755_A/goose/Netherlands/21038413−002/2021
EPI_ISL_19029378_A/Fox/Bavaria/i193/2023
EPI_ISL_15857954_A/laying_hen/Italy/22VIR10267−7/2022
EPI_ISL_17514173_A/black−headed_gull/Austria/23010977−001/2023
EPI_ISL_17971993_A/domestic_cat/Poland/H266−W/2023
EPI_ISL_632316_A/eurasian_curlew/Netherlands/20016890−001/2020
EPI_ISL_16384182_A/greylag_goose/England/119927/2022
EPI_ISL_18728100_A/arctic−fox/Finland/677/2023
EPI_ISL_18122424_A/arctic−fox/Finland/544/2023
EPI_ISL_17699037_A/black−headed_gull_/Sweden/SVA230509SZ0614/FB001431/AB−2023
EPI_ISL_17774358_A/Vulpes_Vulpes/Belgium/03704_0001/2023
EPI_ISL_17821062_A/Black−headed_Gull/Netherlands/77/2023
EPI_ISL_17233302_A/fox/France/23P002844/2023
EPI_ISL_17716819_A/black−headed_gull/Czech_Republic/6947−4/2023
EPI_ISL_17716064_A/black−headed_gull/Austria/23030829−001/2023
EPI_ISL_17245141_A/Vulpes_vulpes/Belgium/01924_0003/2023
EPI_ISL_18115436_A/black−headed_gull/Leningrad_region/RII−WD329S/2023
EPI_ISL_18033223_A/black−headed_gull/Poland/MB180−NMJ/2023
EPI_ISL_17245099_A/Vulpes_vulpes/Belgium/01784_0010/2023
EPI_ISL_18618701_A/mink/Finland/3076/2023
EPI_ISL_18432789_A/peregrine_falcon/Germany−NI/2023AI07594/2023
EPI_ISL_18112326_A/little_tern/England/093351/2023
EPI_ISL_17880914_A/Red_fox/Germany/51023−261/2023
EPI_ISL_18226743_A/red_fox/Northern_Ireland/22185/2023
EPI_ISL_17710043_A/Black−Headed_Gull/England/125550/2023
EPI_ISL_17710034_A/Black−Headed_Gull/England/259311/2023
EPI_ISL_18255293_A/peregrine_falcon/Sweden/SVA230725SZ0382/FB002504/M−2023
EPI_ISL_17982393_A/Sandwich_Tern/Netherlands/7/2023
EPI_ISL_17821064_A/Black−Headed_Gull/Netherlands/79/2023
EPI_ISL_18612177_A/common_tern/Slovenia/PER942−23T2A_23VIR10323−6/2023
EPI_ISL_17679766_A/black−headed_gull/Italy/23VIR1203−4/2023
EPI_ISL_15878541_A/Mink/Spain/3691−2_22VIR10586−8/2022
EPI_ISL_15957660_A/chicken/Wales/138294/2022
EPI_ISL_16904555_A/Red_Fox/Wales/017836/2023
0.005
group
Avian
HPAI in this study
Mammals

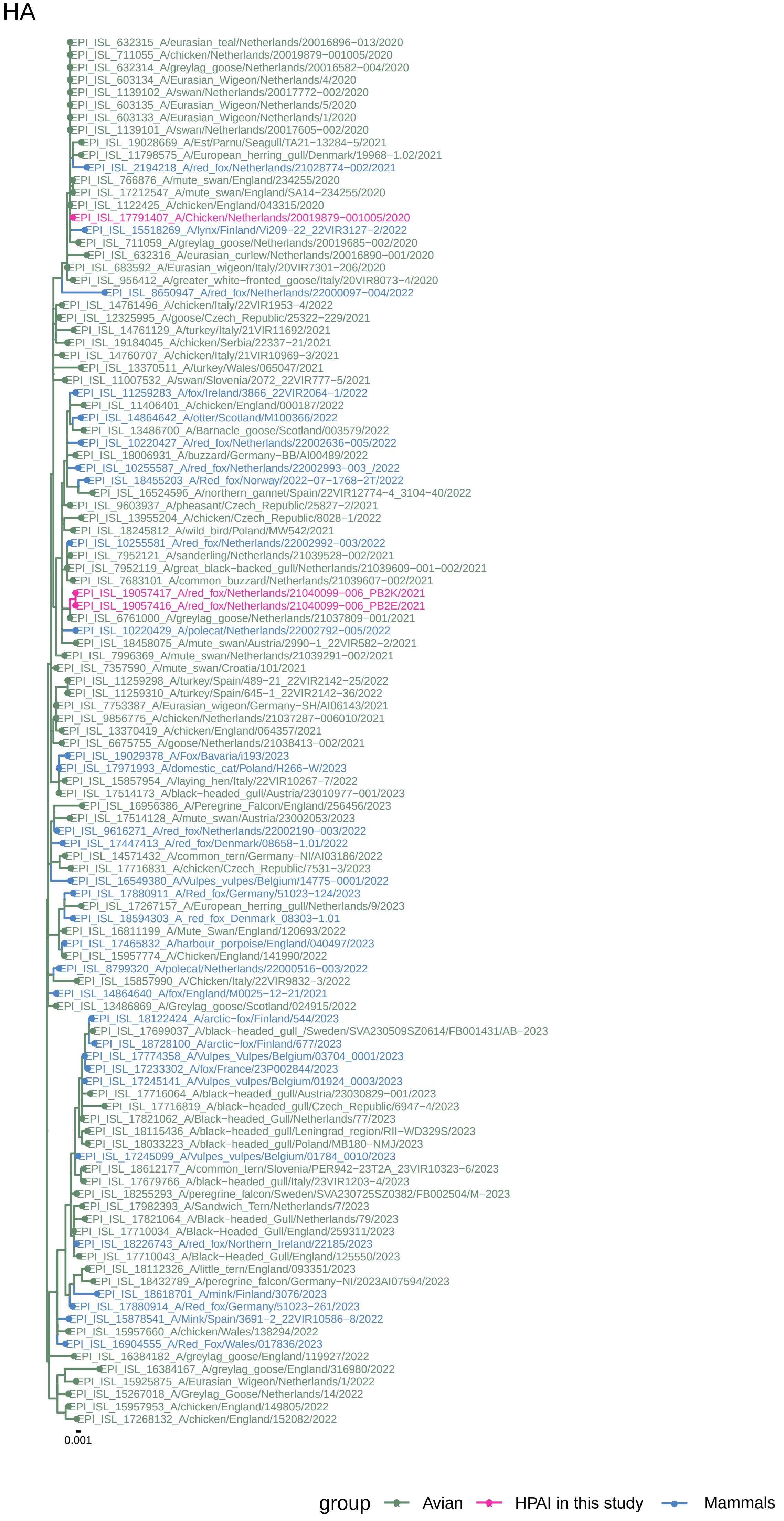
HA
EPI_ISL_632315_A/eurasian_teal/Netherlands/20016896−013/2020
EPI_ISL_711055_A/chicken/Netherlands/20019879−001005/2020
EPI_ISL_632314_A/greylag_goose/Netherlands/20016582−004/2020
EPI_ISL_603134_A/Eurasian_Wigeon/Netherlands/4/2020
EPI_ISL_1139102_A/swan/Netherlands/20017772−002/2020
EPI_ISL_603135_A/Eurasian_Wigeon/Netherlands/5/2020
EPI_ISL_603133_A/Eurasian_Wigeon/Netherlands/1/2020
EPI_ISL_1139101_A/swan/Netherlands/20017605−002/2020
EPI_ISL_19028669_A/Est/Parnu/Seagull/TA21−13284−5/2021
EPI_ISL_11798575_A/European_herring_gull/Denmark/19968−1.02/2021
EPI_ISL_2194218_A/red_fox/Netherlands/21028774−002/2021
EPI_ISL_766876_A/mute_swan/England/234255/2020
EPI_ISL_17212547_A/mute_swan/England/SA14−234255/2020
EPI_ISL_1122425_A/chicken/England/043315/2020
EPI_ISL_17791407_A/Chicken/Netherlands/20019879−001005/2020
EPI_ISL_15518269_A/lynx/Finland/Vi209−22_22VIR3127−2/2022
EPI_ISL_711059_A/greylag_goose/Netherlands/20019685−002/2020
EPI_ISL_632316_A/eurasian_curlew/Netherlands/20016890−001/2020
EPI_ISL_683592_A/Eurasian_wigeon/Italy/20VIR7301−206/2020
EPI_ISL_956412_A/greater_white−fronted_goose/Italy/20VIR8073−4/2020
EPI_ISL_8650947_A/red_fox/Netherlands/22000097−004/2022
EPI_ISL_14761496_A/chicken/Italy/22VIR1953−4/2022
EPI_ISL_12325995_A/goose/Czech_Republic/25322−229/2021
EPI_ISL_14761129_A/turkey/Italy/21VIR11692/2021
EPI_ISL_19184045_A/chicken/Serbia/22337−21/2021
EPI_ISL_14760707_A/chicken/Italy/21VIR10969−3/2021
EPI_ISL_13370511_A/turkey/Wales/065047/2021
EPI_ISL_11007532_A/swan/Slovenia/2072_22VIR777−5/2021
EPI_ISL_11259283_A/fox/Ireland/3866_22VIR2064−1/2022
EPI_ISL_11406401_A/chicken/England/000187/2022
EPI_ISL_14864642_A/otter/Scotland/M100366/2022
EPI_ISL_13486700_A/Barnacle_goose/Scotland/003579/2022
EPI_ISL_10220427_A/red_fox/Netherlands/22002636−005/2022
EPI_ISL_18006931_A/buzzard/Germany−BB/AI00489/2022
EPI_ISL_10255587_A/red_fox/Netherlands/22002993−003_/2022
EPI_ISL_18455203_A/Red_fox/Norway/2022−07−1768−2T/2022
EPI_ISL_16524596_A/northern_gannet/Spain/22VIR12774−4_3104−40/2022
EPI_ISL_9603937_A/pheasant/Czech_Republic/25827−2/2021
EPI_ISL_13955204_A/chicken/Czech_Republic/8028−1/2022
EPI_ISL_18245812_A/wild_bird/Poland/MW542/2021
EPI_ISL_10255581_A/red_fox/Netherlands/22002992−003/2022
EPI_ISL_7952121_A/sanderling/Netherlands/21039528−002/2021
EPI_ISL_7952119_A/great_black−backed_gull/Netherlands/21039609−001−002/2021
EPI_ISL_7683101_A/common_buzzard/Netherlands/21039607−002/2021
EPI_ISL_19057417_A/red_fox/Netherlands/21040099−006_PB2K/2021
EPI_ISL_19057416_A/red_fox/Netherlands/21040099−006_PB2E/2021
EPI_ISL_6761000_A/greylag_goose/Netherlands/21037809−001/2021
EPI_ISL_10220429_A/polecat/Netherlands/22002792−005/2022
EPI_ISL_18458075_A/mute_swan/Austria/2990−1_22VIR582−2/2021
EPI_ISL_7996369_A/mute_swan/Netherlands/21039291−002/2021
EPI_ISL_7357590_A/mute_swan/Croatia/101/2021
EPI_ISL_11259298_A/turkey/Spain/489−21_22VIR2142−25/2022
EPI_ISL_11259310_A/turkey/Spain/645−1_22VIR2142−36/2022
EPI_ISL_7753387_A/Eurasian_wigeon/Germany−SH/AI06143/2021
EPI_ISL_9856775_A/chicken/Netherlands/21037287−006010/2021
EPI_ISL_13370419_A/chicken/England/064357/2021
EPI_ISL_6675755_A/goose/Netherlands/21038413−002/2021
EPI_ISL_19029378_A/Fox/Bavaria/i193/2023
EPI_ISL_17971993_A/domestic_cat/Poland/H266−W/2023
EPI_ISL_15857954_A/laying_hen/Italy/22VIR10267−7/2022
EPI_ISL_17514173_A/black−headed_gull/Austria/23010977−001/2023
EPI_ISL_16956386_A/Peregrine_Falcon/England/256456/2023
EPI_ISL_17514128_A/mute_swan/Austria/23002053/2023
EPI_ISL_9616271_A/red_fox/Netherlands/22002190−003/2022
EPI_ISL_17447413_A/red_fox/Denmark/08658−1.01/2022
EPI_ISL_14571432_A/common_tern/Germany−NI/AI03186/2022
EPI_ISL_17716831_A/chicken/Czech_Republic/7531−3/2023
EPI_ISL_16549380_A/Vulpes_vulpes/Belgium/14775−0001/2022
EPI_ISL_17880911_A/Red_fox/Germany/51023−124/2023
EPI_ISL_17267157_A/European_herring_gull/Netherlands/9/2023
EPI_ISL_18594303_A_red_fox_Denmark_08303−1.01
EPI_ISL_16811199_A/Mute_Swan/England/120693/2022
EPI_ISL_17465832_A/harbour_porpoise/England/040497/2023
EPI_ISL_15957774_A/Chicken/England/141990/2022
EPI_ISL_8799320_A/polecat/Netherlands/22000516−003/2022
EPI_ISL_15857990_A/Chicken/Italy/22VIR9832−3/2022
EPI_ISL_14864640_A/fox/England/M0025−12−21/2021
EPI_ISL_13486869_A/Greylag_goose/Scotland/024915/2022
EPI_ISL_18122424_A/arctic−fox/Finland/544/2023
EPI_ISL_17699037_A/black−headed_gull_/Sweden/SVA230509SZ0614/FB001431/AB−2023
EPI_ISL_18728100_A/arctic−fox/Finland/677/2023
EPI_ISL_17774358_A/Vulpes_Vulpes/Belgium/03704_0001/2023
EPI_ISL_17233302_A/fox/France/23P002844/2023
EPI_ISL_17245141_A/Vulpes_vulpes/Belgium/01924_0003/2023
EPI_ISL_17716064_A/black−headed_gull/Austria/23030829−001/2023
EPI_ISL_17716819_A/black−headed_gull/Czech_Republic/6947−4/2023
EPI_ISL_17821062_A/Black−headed_Gull/Netherlands/77/2023
EPI_ISL_18115436_A/black−headed_gull/Leningrad_region/RII−WD329S/2023
EPI_ISL_18033223_A/black−headed_gull/Poland/MB180−NMJ/2023
EPI_ISL_17245099_A/Vulpes_vulpes/Belgium/01784_0010/2023
EPI_ISL_18612177_A/common_tern/Slovenia/PER942−23T2A_23VIR10323−6/2023
EPI_ISL_17679766_A/black−headed_gull/Italy/23VIR1203−4/2023
EPI_ISL_18255293_A/peregrine_falcon/Sweden/SVA230725SZ0382/FB002504/M−2023
EPI_ISL_17982393_A/Sandwich_Tern/Netherlands/7/2023
EPI_ISL_17821064_A/Black−headed_Gull/Netherlands/79/2023
EPI_ISL_17710034_A/Black−Headed_Gull/England/259311/2023
EPI_ISL_18226743_A/red_fox/Northern_Ireland/22185/2023
EPI_ISL_17710043_A/Black−Headed_Gull/England/125550/2023
EPI_ISL_18112326_A/little_tern/England/093351/2023
EPI_ISL_18432789_A/peregrine_falcon/Germany−NI/2023AI07594/2023
EPI_ISL_18618701_A/mink/Finland/3076/2023
EPI_ISL_17880914_A/Red_fox/Germany/51023−261/2023
EPI_ISL_15878541_A/Mink/Spain/3691−2_22VIR10586−8/2022
EPI_ISL_15957660_A/chicken/Wales/138294/2022
EPI_ISL_16904555_A/Red_Fox/Wales/017836/2023
EPI_ISL_16384182_A/greylag_goose/England/119927/2022
EPI_ISL_16384167_A/greylag_goose/England/316980/2022
EPI_ISL_15925875_A/Eurasian_Wigeon/Netherlands/1/2022
EPI_ISL_15267018_A/Greylag_Goose/Netherlands/14/2022
EPI_ISL_15957953_A/chicken/England/149805/2022
EPI_ISL_17268132_A/chicken/England/152082/2022
0.001
group
Avian
HPAI in this study
Mammals

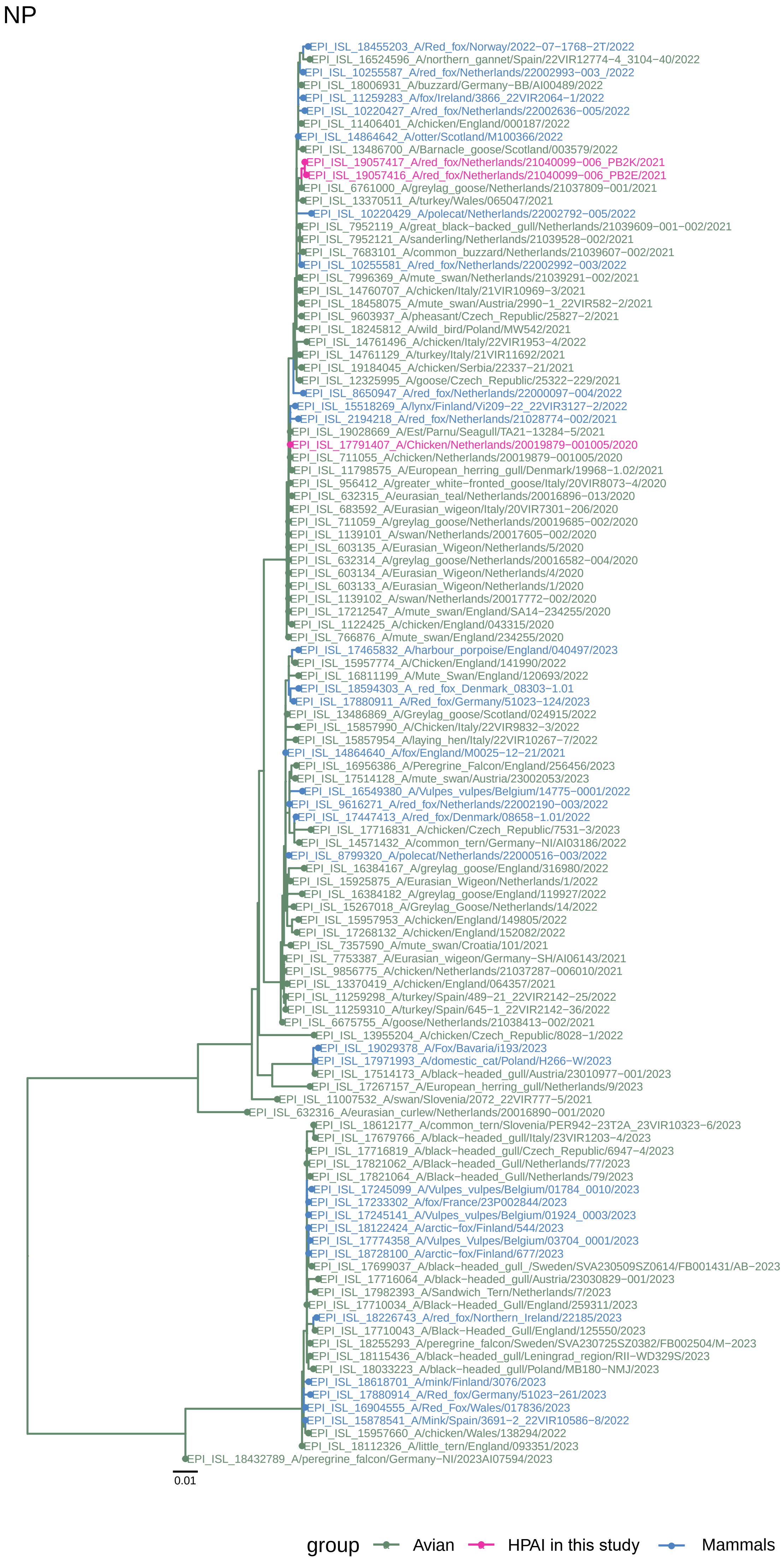
NP
EPI_ISL_18455203_A/Red_fox/Norway/2022−07−1768−2T/2022
EPI_ISL_16524596_A/northern_gannet/Spain/22VIR12774−4_3104−40/2022
EPI_ISL_10255587_A/red_fox/Netherlands/22002993−003_/2022
EPI_ISL_18006931_A/buzzard/Germany−BB/AI00489/2022
EPI_ISL_11259283_A/fox/Ireland/3866_22VIR2064−1/2022
EPI_ISL_10220427_A/red_fox/Netherlands/22002636−005/2022
EPI_ISL_11406401_A/chicken/England/000187/2022
EPI_ISL_14864642_A/otter/Scotland/M100366/2022
EPI_ISL_13486700_A/Barnacle_goose/Scotland/003579/2022
EPI_ISL_19057417_A/red_fox/Netherlands/21040099−006_PB2K/2021
EPI_ISL_19057416_A/red_fox/Netherlands/21040099−006_PB2E/2021
EPI_ISL_6761000_A/greylag_goose/Netherlands/21037809−001/2021
EPI_ISL_13370511_A/turkey/Wales/065047/2021
EPI_ISL_10220429_A/polecat/Netherlands/22002792−005/2022
EPI_ISL_7952119_A/great_black−backed_gull/Netherlands/21039609−001−002/2021
EPI_ISL_7952121_A/sanderling/Netherlands/21039528−002/2021
EPI_ISL_7683101_A/common_buzzard/Netherlands/21039607−002/2021
EPI_ISL_10255581_A/red_fox/Netherlands/22002992−003/2022
EPI_ISL_7996369_A/mute_swan/Netherlands/21039291−002/2021
EPI_ISL_14760707_A/chicken/Italy/21VIR10969−3/2021
EPI_ISL_18458075_A/mute_swan/Austria/2990−1_22VIR582−2/2021
EPI_ISL_9603937_A/pheasant/Czech_Republic/25827−2/2021
EPI_ISL_18245812_A/wild_bird/Poland/MW542/2021
EPI_ISL_14761496_A/chicken/Italy/22VIR1953−4/2022
EPI_ISL_14761129_A/turkey/Italy/21VIR11692/2021
EPI_ISL_19184045_A/chicken/Serbia/22337−21/2021
EPI_ISL_12325995_A/goose/Czech_Republic/25322−229/2021
EPI_ISL_8650947_A/red_fox/Netherlands/22000097−004/2022
EPI_ISL_15518269_A/lynx/Finland/Vi209−22_22VIR3127−2/2022
EPI_ISL_2194218_A/red_fox/Netherlands/21028774−002/2021
EPI_ISL_19028669_A/Est/Parnu/Seagull/TA21−13284−5/2021
EPI_ISL_17791407_A/Chicken/Netherlands/20019879−001005/2020
EPI_ISL_711055_A/chicken/Netherlands/20019879−001005/2020
EPI_ISL_11798575_A/European_herring_gull/Denmark/19968−1.02/2021
EPI_ISL_956412_A/greater_white−fronted_goose/Italy/20VIR8073−4/2020
EPI_ISL_632315_A/eurasian_teal/Netherlands/20016896−013/2020
EPI_ISL_683592_A/Eurasian_wigeon/Italy/20VIR7301−206/2020
EPI_ISL_711059_A/greylag_goose/Netherlands/20019685−002/2020
EPI_ISL_1139101_A/swan/Netherlands/20017605−002/2020
EPI_ISL_603135_A/Eurasian_Wigeon/Netherlands/5/2020
EPI_ISL_632314_A/greylag_goose/Netherlands/20016582−004/2020
EPI_ISL_603134_A/Eurasian_Wigeon/Netherlands/4/2020
EPI_ISL_603133_A/Eurasian_Wigeon/Netherlands/1/2020
EPI_ISL_1139102_A/swan/Netherlands/20017772−002/2020
EPI_ISL_17212547_A/mute_swan/England/SA14−234255/2020
EPI_ISL_1122425_A/chicken/England/043315/2020
EPI_ISL_766876_A/mute_swan/England/234255/2020
EPI_ISL_17465832_A/harbour_porpoise/England/040497/2023
EPI_ISL_15957774_A/Chicken/England/141990/2022
EPI_ISL_16811199_A/Mute_Swan/England/120693/2022
EPI_ISL_18594303_A_red_fox_Denmark_08303−1.01
EPI_ISL_17880911_A/Red_fox/Germany/51023−124/2023
EPI_ISL_13486869_A/Greylag_goose/Scotland/024915/2022
EPI_ISL_15857990_A/Chicken/Italy/22VIR9832−3/2022
EPI_ISL_15857954_A/laying_hen/Italy/22VIR10267−7/2022
EPI_ISL_14864640_A/fox/England/M0025−12−21/2021
EPI_ISL_16956386_A/Peregrine_Falcon/England/256456/2023
EPI_ISL_17514128_A/mute_swan/Austria/23002053/2023
EPI_ISL_16549380_A/Vulpes_vulpes/Belgium/14775−0001/2022
EPI_ISL_9616271_A/red_fox/Netherlands/22002190−003/2022
EPI_ISL_17447413_A/red_fox/Denmark/08658−1.01/2022
EPI_ISL_17716831_A/chicken/Czech_Republic/7531−3/2023
EPI_ISL_14571432_A/common_tern/Germany−NI/AI03186/2022
EPI_ISL_8799320_A/polecat/Netherlands/22000516−003/2022
EPI_ISL_16384167_A/greylag_goose/England/316980/2022
EPI_ISL_15925875_A/Eurasian_Wigeon/Netherlands/1/2022
EPI_ISL_16384182_A/greylag_goose/England/119927/2022
EPI_ISL_15267018_A/Greylag_Goose/Netherlands/14/2022
EPI_ISL_15957953_A/chicken/England/149805/2022
EPI_ISL_17268132_A/chicken/England/152082/2022
EPI_ISL_7357590_A/mute_swan/Croatia/101/2021
EPI_ISL_7753387_A/Eurasian_wigeon/Germany−SH/AI06143/2021
EPI_ISL_9856775_A/chicken/Netherlands/21037287−006010/2021
EPI_ISL_13370419_A/chicken/England/064357/2021
EPI_ISL_11259298_A/turkey/Spain/489−21_22VIR2142−25/2022
EPI_ISL_11259310_A/turkey/Spain/645−1_22VIR2142−36/2022
EPI_ISL_6675755_A/goose/Netherlands/21038413−002/2021
EPI_ISL_13955204_A/chicken/Czech_Republic/8028−1/2022
EPI_ISL_19029378_A/Fox/Bavaria/i193/2023
EPI_ISL_17971993_A/domestic_cat/Poland/H266−W/2023
EPI_ISL_17514173_A/black−headed_gull/Austria/23010977−001/2023
EPI_ISL_17267157_A/European_herring_gull/Netherlands/9/2023
EPI_ISL_11007532_A/swan/Slovenia/2072_22VIR777−5/2021
EPI_ISL_632316_A/eurasian_curlew/Netherlands/20016890−001/2020
EPI_ISL_18612177_A/common_tern/Slovenia/PER942−23T2A_23VIR10323−6/2023
EPI_ISL_17679766_A/black−headed_gull/Italy/23VIR1203−4/2023
EPI_ISL_17716819_A/black−headed_gull/Czech_Republic/6947−4/2023
EPI_ISL_17821062_A/Black−headed_Gull/Netherlands/77/2023
EPI_ISL_17821064_A/Black−headed_Gull/Netherlands/79/2023
EPI_ISL_17245099_A/Vulpes_vulpes/Belgium/01784_0010/2023
EPI_ISL_17233302_A/fox/France/23P002844/2023
EPI_ISL_17245141_A/Vulpes_vulpes/Belgium/01924_0003/2023
EPI_ISL_18122424_A/arctic−fox/Finland/544/2023
EPI_ISL_17774358_A/Vulpes_Vulpes/Belgium/03704_0001/2023
EPI_ISL_18728100_A/arctic−fox/Finland/677/2023
EPI_ISL_17699037_A/black−headed_gull_/Sweden/SVA230509SZ0614/FB001431/AB−2023
EPI_ISL_17716064_A/black−headed_gull/Austria/23030829−001/2023
EPI_ISL_17982393_A/Sandwich_Tern/Netherlands/7/2023
EPI_ISL_17710034_A/Black−Headed_Gull/England/259311/2023
EPI_ISL_18226743_A/red_fox/Northern_Ireland/22185/2023
EPI_ISL_17710043_A/Black−Headed_Gull/England/125550/2023
EPI_ISL_18255293_A/peregrine_falcon/Sweden/SVA230725SZ0382/FB002504/M−2023
EPI_ISL_18115436_A/black−headed_gull/Leningrad_region/RII−WD329S/2023
EPI_ISL_18033223_A/black−headed_gull/Poland/MB180−NMJ/2023
EPI_ISL_18618701_A/mink/Finland/3076/2023
EPI_ISL_17880914_A/Red_fox/Germany/51023−261/2023
EPI_ISL_16904555_A/Red_Fox/Wales/017836/2023
EPI_ISL_15878541_A/Mink/Spain/3691−2_22VIR10586−8/2022
EPI_ISL_15957660_A/chicken/Wales/138294/2022
EPI_ISL_18112326_A/little_tern/England/093351/2023
EPI_ISL_18432789_A/peregrine_falcon/Germany−NI/2023AI07594/2023
0.01
group
Avian
HPAI in this study
Mammals

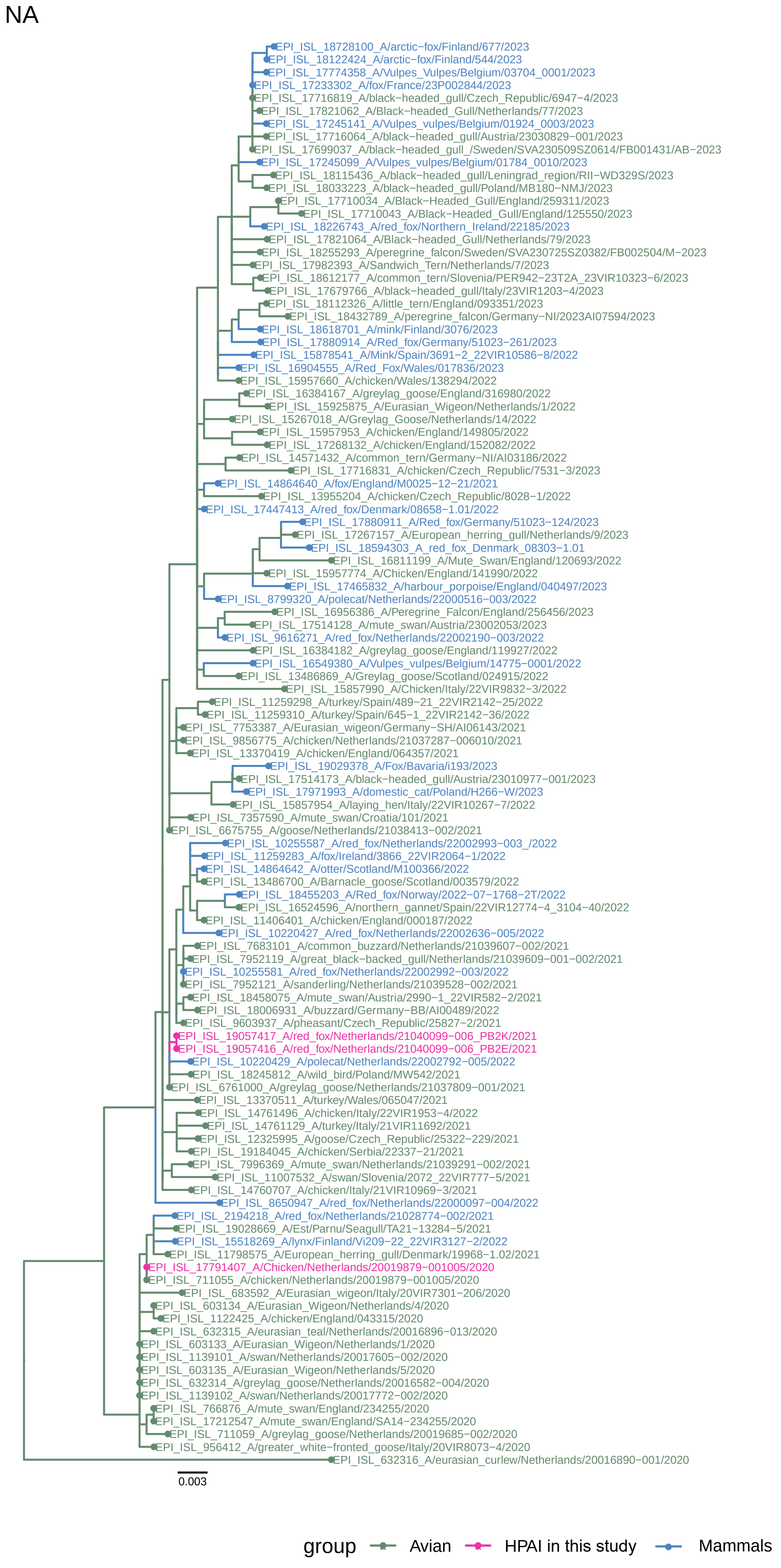
NA
EPI_ISL_18728100_A/arctic−fox/Finland/677/2023
EPI_ISL_18122424_A/arctic−fox/Finland/544/2023
EPI_ISL_17774358_A/Vulpes_Vulpes/Belgium/03704_0001/2023
EPI_ISL_17233302_A/fox/France/23P002844/2023
EPI_ISL_17716819_A/black−headed_gull/Czech_Republic/6947−4/2023
EPI_ISL_17821062_A/Black−headed_Gull/Netherlands/77/2023
EPI_ISL_17245141_A/Vulpes_vulpes/Belgium/01924_0003/2023
EPI_ISL_17716064_A/black−headed_gull/Austria/23030829−001/2023
EPI_ISL_17699037_A/black−headed_gull_/Sweden/SVA230509SZ0614/FB001431/AB−2023
EPI_ISL_17245099_A/Vulpes_vulpes/Belgium/01784_0010/2023
EPI_ISL_18115436_A/black−headed_gull/Leningrad_region/RII−WD329S/2023
EPI_ISL_18033223_A/black−headed_gull/Poland/MB180−NMJ/2023
EPI_ISL_17710034_A/Black−Headed_Gull/England/259311/2023
EPI_ISL_17710043_A/Black−Headed_Gull/England/125550/2023
EPI_ISL_18226743_A/red_fox/Northern_Ireland/22185/2023
EPI_ISL_17821064_A/Black−headed_Gull/Netherlands/79/2023
EPI_ISL_18255293_A/peregrine_falcon/Sweden/SVA230725SZ0382/FB002504/M−2023
EPI_ISL_17982393_A/Sandwich_Tern/Netherlands/7/2023
EPI_ISL_18612177_A/common_tern/Slovenia/PER942−23T2A_23VIR10323−6/2023
EPI_ISL_17679766_A/black−headed_gull/Italy/23VIR1203−4/2023
EPI_ISL_18112326_A/little_tern/England/093351/2023
EPI_ISL_18432789_A/peregrine_falcon/Germany−NI/2023AI07594/2023
EPI_ISL_18618701_A/mink/Finland/3076/2023
EPI_ISL_17880914_A/Red_fox/Germany/51023−261/2023
EPI_ISL_15878541_A/Mink/Spain/3691−2_22VIR10586−8/2022
EPI_ISL_16904555_A/Red_Fox/Wales/017836/2023
EPI_ISL_15957660_A/chicken/Wales/138294/2022
EPI_ISL_16384167_A/greylag_goose/England/316980/2022
EPI_ISL_15925875_A/Eurasian_Wigeon/Netherlands/1/2022
EPI_ISL_15267018_A/Greylag_Goose/Netherlands/14/2022
EPI_ISL_15957953_A/chicken/England/149805/2022
EPI_ISL_17268132_A/chicken/England/152082/2022
EPI_ISL_14571432_A/common_tern/Germany−NI/AI03186/2022
EPI_ISL_17716831_A/chicken/Czech_Republic/7531−3/2023
EPI_ISL_14864640_A/fox/England/M0025−12−21/2021
EPI_ISL_13955204_A/chicken/Czech_Republic/8028−1/2022
EPI_ISL_17447413_A/red_fox/Denmark/08658−1.01/2022
EPI_ISL_17880911_A/Red_fox/Germany/51023−124/2023
EPI_ISL_17267157_A/European_herring_gull/Netherlands/9/2023
EPI_ISL_18594303_A_red_fox_Denmark_08303−1.01
EPI_ISL_16811199_A/Mute_Swan/England/120693/2022
EPI_ISL_15957774_A/Chicken/England/141990/2022
EPI_ISL_17465832_A/harbour_porpoise/England/040497/2023
EPI_ISL_8799320_A/polecat/Netherlands/22000516−003/2022
EPI_ISL_16956386_A/Peregrine_Falcon/England/256456/2023
EPI_ISL_17514128_A/mute_swan/Austria/23002053/2023
EPI_ISL_9616271_A/red_fox/Netherlands/22002190−003/2022
EPI_ISL_16384182_A/greylag_goose/England/119927/2022
EPI_ISL_16549380_A/Vulpes_vulpes/Belgium/14775−0001/2022
EPI_ISL_13486869_A/Greylag_goose/Scotland/024915/2022
EPI_ISL_15857990_A/Chicken/Italy/22VIR9832−3/2022
EPI_ISL_11259298_A/turkey/Spain/489−21_22VIR2142−25/2022
EPI_ISL_11259310_A/turkey/Spain/645−1_22VIR2142−36/2022
EPI_ISL_7753387_A/Eurasian_wigeon/Germany−SH/AI06143/2021
EPI_ISL_9856775_A/chicken/Netherlands/21037287−006010/2021
EPI_ISL_13370419_A/chicken/England/064357/2021
EPI_ISL_19029378_A/Fox/Bavaria/i193/2023
EPI_ISL_17514173_A/black−headed_gull/Austria/23010977−001/2023
EPI_ISL_17971993_A/domestic_cat/Poland/H266−W/2023
EPI_ISL_15857954_A/laying_hen/Italy/22VIR10267−7/2022
EPI_ISL_7357590_A/mute_swan/Croatia/101/2021
EPI_ISL_6675755_A/goose/Netherlands/21038413−002/2021
EPI_ISL_10255587_A/red_fox/Netherlands/22002993−003_/2022
EPI_ISL_11259283_A/fox/Ireland/3866_22VIR2064−1/2022
EPI_ISL_14864642_A/otter/Scotland/M100366/2022
EPI_ISL_13486700_A/Barnacle_goose/Scotland/003579/2022
EPI_ISL_18455203_A/Red_fox/Norway/2022−07−1768−2T/2022
EPI_ISL_16524596_A/northern_gannet/Spain/22VIR12774−4_3104−40/2022
EPI_ISL_11406401_A/chicken/England/000187/2022
EPI_ISL_10220427_A/red_fox/Netherlands/22002636−005/2022
EPI_ISL_7683101_A/common_buzzard/Netherlands/21039607−002/2021
EPI_ISL_7952119_A/great_black−backed_gull/Netherlands/21039609−001−002/2021
EPI_ISL_10255581_A/red_fox/Netherlands/22002992−003/2022
EPI_ISL_7952121_A/sanderling/Netherlands/21039528−002/2021
EPI_ISL_18458075_A/mute_swan/Austria/2990−1_22VIR582−2/2021
EPI_ISL_18006931_A/buzzard/Germany−BB/AI00489/2022
EPI_ISL_9603937_A/pheasant/Czech_Republic/25827−2/2021
EPI_ISL_19057417_A/red_fox/Netherlands/21040099−006_PB2K/2021
EPI_ISL_19057416_A/red_fox/Netherlands/21040099−006_PB2E/2021
EPI_ISL_10220429_A/polecat/Netherlands/22002792−005/2022
EPI_ISL_18245812_A/wild_bird/Poland/MW542/2021
EPI_ISL_6761000_A/greylag_goose/Netherlands/21037809−001/2021
EPI_ISL_13370511_A/turkey/Wales/065047/2021
EPI_ISL_14761496_A/chicken/Italy/22VIR1953−4/2022
EPI_ISL_14761129_A/turkey/Italy/21VIR11692/2021
EPI_ISL_12325995_A/goose/Czech_Republic/25322−229/2021
EPI_ISL_19184045_A/chicken/Serbia/22337−21/2021
EPI_ISL_7996369_A/mute_swan/Netherlands/21039291−002/2021
EPI_ISL_11007532_A/swan/Slovenia/2072_22VIR777−5/2021
EPI_ISL_14760707_A/chicken/Italy/21VIR10969−3/2021
EPI_ISL_8650947_A/red_fox/Netherlands/22000097−004/2022
EPI_ISL_2194218_A/red_fox/Netherlands/21028774−002/2021
EPI_ISL_19028669_A/Est/Parnu/Seagull/TA21−13284−5/2021
EPI_ISL_15518269_A/lynx/Finland/Vi209−22_22VIR3127−2/2022
EPI_ISL_11798575_A/European_herring_gull/Denmark/19968−1.02/2021
EPI_ISL_17791407_A/Chicken/Netherlands/20019879−001005/2020
EPI_ISL_711055_A/chicken/Netherlands/20019879−001005/2020
EPI_ISL_683592_A/Eurasian_wigeon/Italy/20VIR7301−206/2020
EPI_ISL_603134_A/Eurasian_Wigeon/Netherlands/4/2020
EPI_ISL_1122425_A/chicken/England/043315/2020
EPI_ISL_632315_A/eurasian_teal/Netherlands/20016896−013/2020
EPI_ISL_603133_A/Eurasian_Wigeon/Netherlands/1/2020
EPI_ISL_1139101_A/swan/Netherlands/20017605−002/2020
EPI_ISL_603135_A/Eurasian_Wigeon/Netherlands/5/2020
EPI_ISL_632314_A/greylag_goose/Netherlands/20016582−004/2020
EPI_ISL_1139102_A/swan/Netherlands/20017772−002/2020
EPI_ISL_766876_A/mute_swan/England/234255/2020
EPI_ISL_17212547_A/mute_swan/England/SA14−234255/2020
EPI_ISL_711059_A/greylag_goose/Netherlands/20019685−002/2020
EPI_ISL_956412_A/greater_white−fronted_goose/Italy/20VIR8073−4/2020
EPI_ISL_632316_A/eurasian_curlew/Netherlands/20016890−001/2020
0.003
group
Avian
HPAI in this study
Mammals

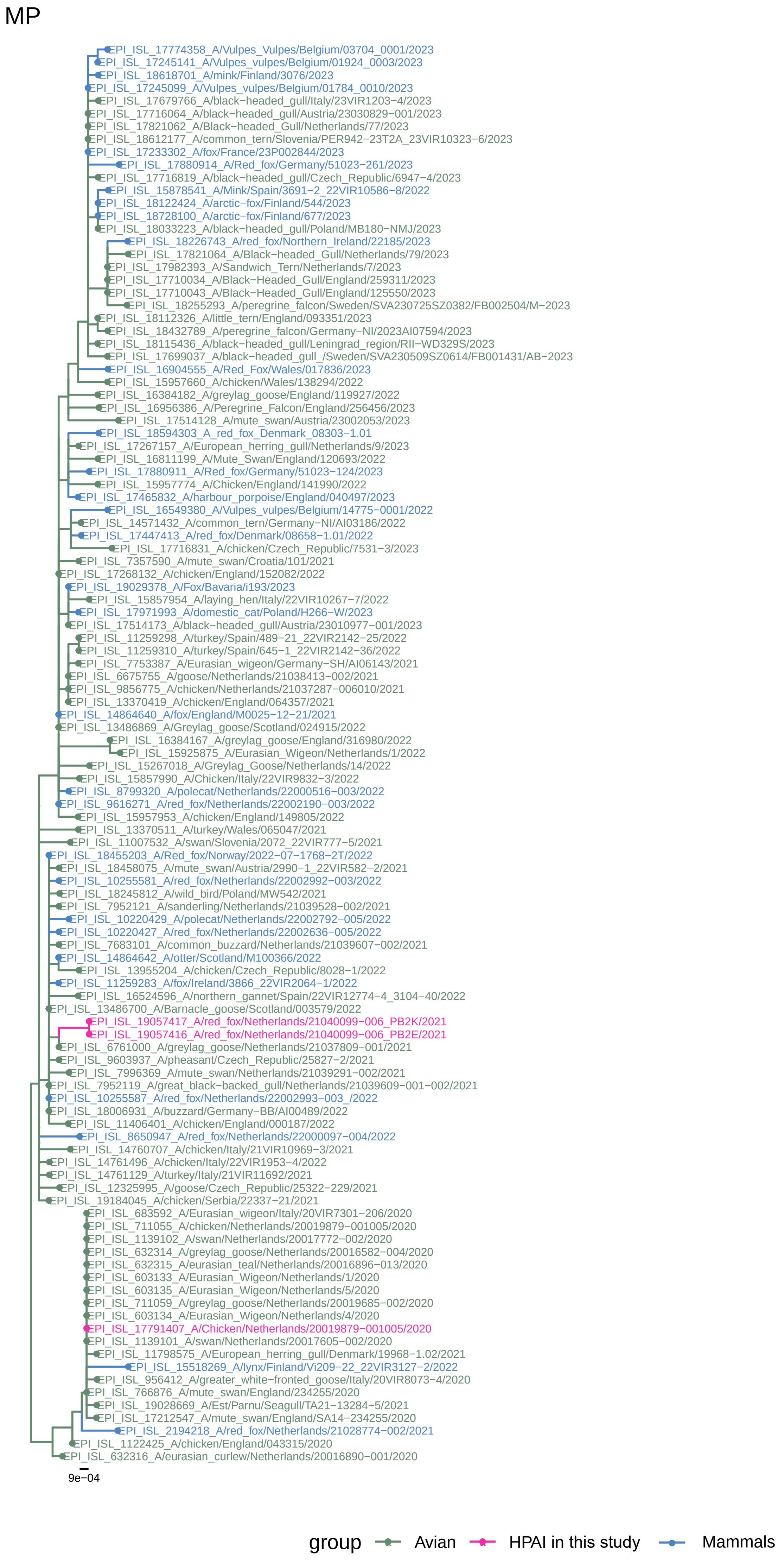
MP
EPI_ISL_17774358_A/Vulpes_Vulpes/Belgium/03704_0001/2023
EPI_ISL_17245141_A/Vulpes_vulpes/Belgium/01924_0003/2023
EPI_ISL_18618701_A/mink/Finland/3076/2023
EPI_ISL_17245099_A/Vulpes_vulpes/Belgium/01784_0010/2023
EPI_ISL_17679766_A/black−headed_gull/Italy/23VIR1203−4/2023
EPI_ISL_17716064_A/black−headed_gull/Austria/23030829−001/2023
EPI_ISL_17821062_A/Black−headed_Gull/Netherlands/77/2023
EPI_ISL_18612177_A/common_tern/Slovenia/PER942−23T2A_23VIR10323−6/2023
EPI_ISL_17233302_A/fox/France/23P002844/2023
EPI_ISL_17880914_A/Red_fox/Germany/51023−261/2023
EPI_ISL_17716819_A/black−headed_gull/Czech_Republic/6947−4/2023
EPI_ISL_15878541_A/Mink/Spain/3691−2_22VIR10586−8/2022
EPI_ISL_18122424_A/arctic−fox/Finland/544/2023
EPI_ISL_18728100_A/arctic−fox/Finland/677/2023
EPI_ISL_18033223_A/black−headed_gull/Poland/MB180−NMJ/2023
EPI_ISL_18226743_A/red_fox/Northern_Ireland/22185/2023
EPI_ISL_17821064_A/Black−headed_Gull/Netherlands/79/2023
EPI_ISL_17982393_A/Sandwich_Tern/Netherlands/7/2023
EPI_ISL_17710034_A/Black−Headed_Gull/England/259311/2023
EPI_ISL_17710043_A/Black−Headed_Gull/England/125550/2023
EPI_ISL_18255293_A/peregrine_falcon/Sweden/SVA230725SZ0382/FB002504/M−2023
EPI_ISL_18112326_A/little_tern/England/093351/2023
EPI_ISL_18432789_A/peregrine_falcon/Germany−NI/2023AI07594/2023
EPI_ISL_18115436_A/black−headed_gull/Leningrad_region/RII−WD329S/2023
EPI_ISL_17699037_A/black−headed_gull_/Sweden/SVA230509SZ0614/FB001431/AB−2023
EPI_ISL_16904555_A/Red_Fox/Wales/017836/2023
EPI_ISL_15957660_A/chicken/Wales/138294/2022
EPI_ISL_16384182_A/greylag_goose/England/119927/2022
EPI_ISL_16956386_A/Peregrine_Falcon/England/256456/2023
EPI_ISL_17514128_A/mute_swan/Austria/23002053/2023
EPI_ISL_18594303_A_red_fox_Denmark_08303−1.01
EPI_ISL_17267157_A/European_herring_gull/Netherlands/9/2023
EPI_ISL_16811199_A/Mute_Swan/England/120693/2022
EPI_ISL_17880911_A/Red_fox/Germany/51023−124/2023
EPI_ISL_15957774_A/Chicken/England/141990/2022
EPI_ISL_17465832_A/harbour_porpoise/England/040497/2023
EPI_ISL_16549380_A/Vulpes_vulpes/Belgium/14775−0001/2022
EPI_ISL_14571432_A/common_tern/Germany−NI/AI03186/2022
EPI_ISL_17447413_A/red_fox/Denmark/08658−1.01/2022
EPI_ISL_17716831_A/chicken/Czech_Republic/7531−3/2023
EPI_ISL_7357590_A/mute_swan/Croatia/101/2021
EPI_ISL_17268132_A/chicken/England/152082/2022
EPI_ISL_19029378_A/Fox/Bavaria/i193/2023
EPI_ISL_15857954_A/laying_hen/Italy/22VIR10267−7/2022
EPI_ISL_17971993_A/domestic_cat/Poland/H266−W/2023
EPI_ISL_17514173_A/black−headed_gull/Austria/23010977−001/2023
EPI_ISL_11259298_A/turkey/Spain/489−21_22VIR2142−25/2022
EPI_ISL_11259310_A/turkey/Spain/645−1_22VIR2142−36/2022
EPI_ISL_7753387_A/Eurasian_wigeon/Germany−SH/AI06143/2021
EPI_ISL_6675755_A/goose/Netherlands/21038413−002/2021
EPI_ISL_9856775_A/chicken/Netherlands/21037287−006010/2021
EPI_ISL_13370419_A/chicken/England/064357/2021
EPI_ISL_14864640_A/fox/England/M0025−12−21/2021
EPI_ISL_13486869_A/Greylag_goose/Scotland/024915/2022
EPI_ISL_16384167_A/greylag_goose/England/316980/2022
EPI_ISL_15925875_A/Eurasian_Wigeon/Netherlands/1/2022
EPI_ISL_15267018_A/Greylag_Goose/Netherlands/14/2022
EPI_ISL_15857990_A/Chicken/Italy/22VIR9832−3/2022
EPI_ISL_8799320_A/polecat/Netherlands/22000516−003/2022
EPI_ISL_9616271_A/red_fox/Netherlands/22002190−003/2022
EPI_ISL_15957953_A/chicken/England/149805/2022
EPI_ISL_13370511_A/turkey/Wales/065047/2021
EPI_ISL_11007532_A/swan/Slovenia/2072_22VIR777−5/2021
EPI_ISL_18455203_A/Red_fox/Norway/2022−07−1768−2T/2022
EPI_ISL_18458075_A/mute_swan/Austria/2990−1_22VIR582−2/2021
EPI_ISL_10255581_A/red_fox/Netherlands/22002992−003/2022
EPI_ISL_18245812_A/wild_bird/Poland/MW542/2021
EPI_ISL_7952121_A/sanderling/Netherlands/21039528−002/2021
EPI_ISL_10220429_A/polecat/Netherlands/22002792−005/2022
EPI_ISL_10220427_A/red_fox/Netherlands/22002636−005/2022
EPI_ISL_7683101_A/common_buzzard/Netherlands/21039607−002/2021
EPI_ISL_14864642_A/otter/Scotland/M100366/2022
EPI_ISL_13955204_A/chicken/Czech_Republic/8028−1/2022
EPI_ISL_11259283_A/fox/Ireland/3866_22VIR2064−1/2022
EPI_ISL_16524596_A/northern_gannet/Spain/22VIR12774−4_3104−40/2022
EPI_ISL_13486700_A/Barnacle_goose/Scotland/003579/2022
EPI_ISL_19057417_A/red_fox/Netherlands/21040099−006_PB2K/2021
EPI_ISL_19057416_A/red_fox/Netherlands/21040099−006_PB2E/2021
EPI_ISL_6761000_A/greylag_goose/Netherlands/21037809−001/2021
EPI_ISL_9603937_A/pheasant/Czech_Republic/25827−2/2021
EPI_ISL_7996369_A/mute_swan/Netherlands/21039291−002/2021
EPI_ISL_7952119_A/great_black−backed_gull/Netherlands/21039609−001−002/2021
EPI_ISL_10255587_A/red_fox/Netherlands/22002993−003_/2022
EPI_ISL_18006931_A/buzzard/Germany−BB/AI00489/2022
EPI_ISL_11406401_A/chicken/England/000187/2022
EPI_ISL_8650947_A/red_fox/Netherlands/22000097−004/2022
EPI_ISL_14760707_A/chicken/Italy/21VIR10969−3/2021
EPI_ISL_14761496_A/chicken/Italy/22VIR1953−4/2022
EPI_ISL_14761129_A/turkey/Italy/21VIR11692/2021
EPI_ISL_12325995_A/goose/Czech_Republic/25322−229/2021
EPI_ISL_19184045_A/chicken/Serbia/22337−21/2021
EPI_ISL_683592_A/Eurasian_wigeon/Italy/20VIR7301−206/2020
EPI_ISL_711055_A/chicken/Netherlands/20019879−001005/2020
EPI_ISL_1139102_A/swan/Netherlands/20017772−002/2020
EPI_ISL_632314_A/greylag_goose/Netherlands/20016582−004/2020
EPI_ISL_632315_A/eurasian_teal/Netherlands/20016896−013/2020
EPI_ISL_603133_A/Eurasian_Wigeon/Netherlands/1/2020
EPI_ISL_603135_A/Eurasian_Wigeon/Netherlands/5/2020
EPI_ISL_711059_A/greylag_goose/Netherlands/20019685−002/2020
EPI_ISL_603134_A/Eurasian_Wigeon/Netherlands/4/2020
EPI_ISL_17791407_A/Chicken/Netherlands/20019879−001005/2020
EPI_ISL_1139101_A/swan/Netherlands/20017605−002/2020
EPI_ISL_11798575_A/European_herring_gull/Denmark/19968−1.02/2021
EPI_ISL_15518269_A/lynx/Finland/Vi209−22_22VIR3127−2/2022
EPI_ISL_956412_A/greater_white−fronted_goose/Italy/20VIR8073−4/2020
EPI_ISL_766876_A/mute_swan/England/234255/2020
EPI_ISL_19028669_A/Est/Parnu/Seagull/TA21−13284−5/2021
EPI_ISL_17212547_A/mute_swan/England/SA14−234255/2020
EPI_ISL_2194218_A/red_fox/Netherlands/21028774−002/2021
EPI_ISL_1122425_A/chicken/England/043315/2020
EPI_ISL_632316_A/eurasian_curlew/Netherlands/20016890−001/2020
9e−04
group
Avian
HPAI in this study
Mammals

NS

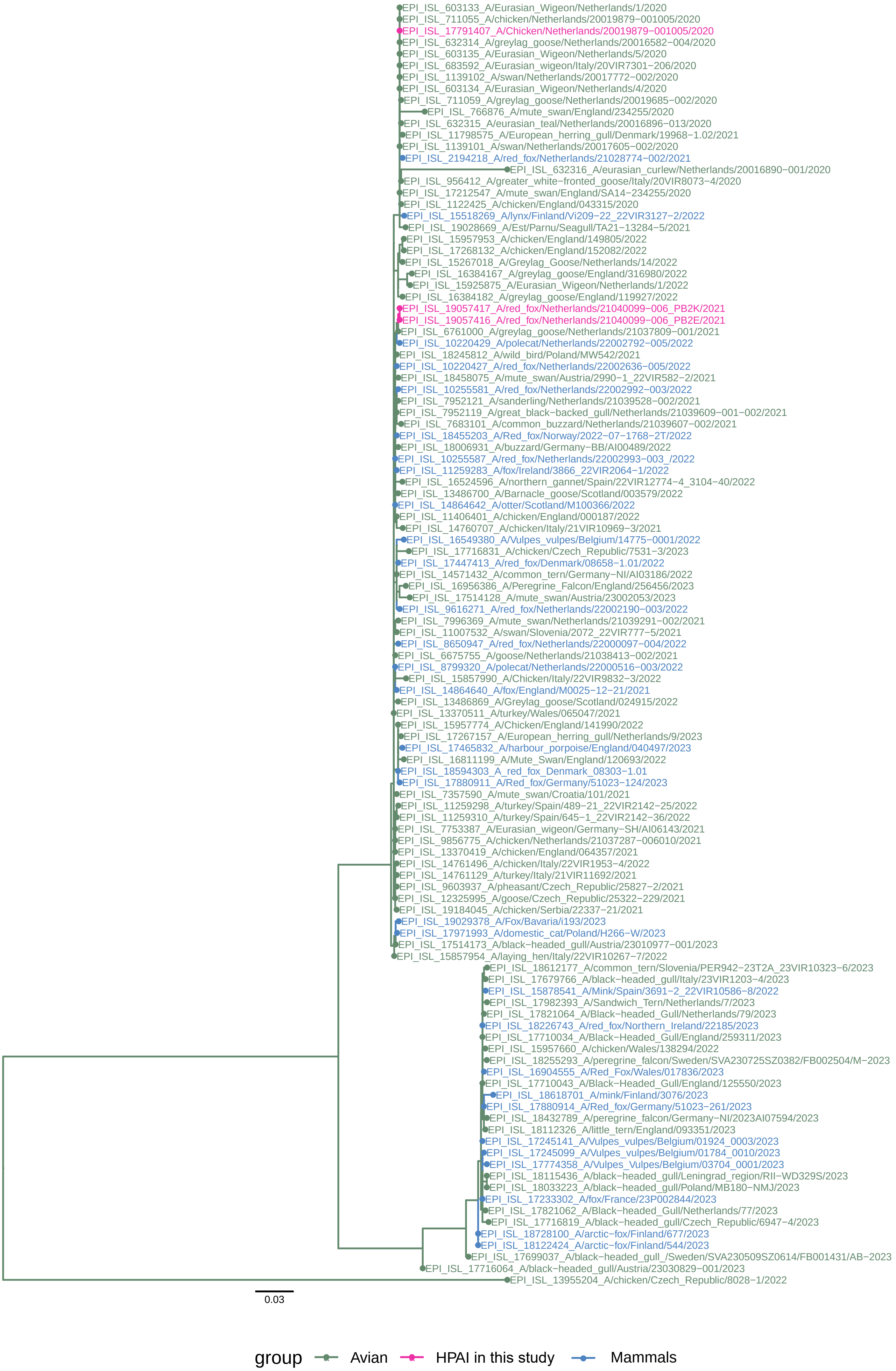

EPI_ISL_603133_A/Eurasian_Wigeon/Netherlands/1/2020
EPI_ISL_711055_A/chicken/Netherlands/20019879−001005/2020
EPI_ISL_17791407_A/Chicken/Netherlands/20019879−001005/2020
EPI_ISL_632314_A/greylag_goose/Netherlands/20016582−004/2020
EPI_ISL_603135_A/Eurasian_Wigeon/Netherlands/5/2020
EPI_ISL_683592_A/Eurasian_wigeon/Italy/20VIR7301−206/2020
EPI_ISL_1139102_A/swan/Netherlands/20017772−002/2020
EPI_ISL_603134_A/Eurasian_Wigeon/Netherlands/4/2020
EPI_ISL_711059_A/greylag_goose/Netherlands/20019685−002/2020
EPI_ISL_766876_A/mute_swan/England/234255/2020
EPI_ISL_632315_A/eurasian_teal/Netherlands/20016896−013/2020
EPI_ISL_11798575_A/European_herring_gull/Denmark/19968−1.02/2021
EPI_ISL_1139101_A/swan/Netherlands/20017605−002/2020
EPI_ISL_2194218_A/red_fox/Netherlands/21028774−002/2021
EPI_ISL_632316_A/eurasian_curlew/Netherlands/20016890−001/2020
EPI_ISL_956412_A/greater_white−fronted_goose/Italy/20VIR8073−4/2020
EPI_ISL_17212547_A/mute_swan/England/SA14−234255/2020
EPI_ISL_1122425_A/chicken/England/043315/2020
EPI_ISL_15518269_A/lynx/Finland/Vi209−22_22VIR3127−2/2022
EPI_ISL_19028669_A/Est/Parnu/Seagull/TA21−13284−5/2021
EPI_ISL_15957953_A/chicken/England/149805/2022
EPI_ISL_17268132_A/chicken/England/152082/2022
EPI_ISL_15267018_A/Greylag_Goose/Netherlands/14/2022
EPI_ISL_16384167_A/greylag_goose/England/316980/2022
EPI_ISL_15925875_A/Eurasian_Wigeon/Netherlands/1/2022
EPI_ISL_16384182_A/greylag_goose/England/119927/2022
EPI_ISL_19057417_A/red_fox/Netherlands/21040099−006_PB2K/2021
EPI_ISL_19057416_A/red_fox/Netherlands/21040099−006_PB2E/2021
EPI_ISL_6761000_A/greylag_goose/Netherlands/21037809−001/2021
EPI_ISL_10220429_A/polecat/Netherlands/22002792−005/2022
EPI_ISL_18245812_A/wild_bird/Poland/MW542/2021
EPI_ISL_10220427_A/red_fox/Netherlands/22002636−005/2022
EPI_ISL_18458075_A/mute_swan/Austria/2990−1_22VIR582−2/2021
EPI_ISL_10255581_A/red_fox/Netherlands/22002992−003/2022
EPI_ISL_7952121_A/sanderling/Netherlands/21039528−002/2021
EPI_ISL_7952119_A/great_black−backed_gull/Netherlands/21039609−001−002/2021
EPI_ISL_7683101_A/common_buzzard/Netherlands/21039607−002/2021
EPI_ISL_18455203_A/Red_fox/Norway/2022−07−1768−2T/2022
EPI_ISL_18006931_A/buzzard/Germany−BB/AI00489/2022
EPI_ISL_10255587_A/red_fox/Netherlands/22002993−003_/2022
EPI_ISL_11259283_A/fox/Ireland/3866_22VIR2064−1/2022
EPI_ISL_16524596_A/northern_gannet/Spain/22VIR12774−4_3104−40/2022
EPI_ISL_13486700_A/Barnacle_goose/Scotland/003579/2022
EPI_ISL_14864642_A/otter/Scotland/M100366/2022
EPI_ISL_11406401_A/chicken/England/000187/2022
EPI_ISL_14760707_A/chicken/Italy/21VIR10969−3/2021
EPI_ISL_16549380_A/Vulpes_vulpes/Belgium/14775−0001/2022
EPI_ISL_17716831_A/chicken/Czech_Republic/7531−3/2023
EPI_ISL_17447413_A/red_fox/Denmark/08658−1.01/2022
EPI_ISL_14571432_A/common_tern/Germany−NI/AI03186/2022
EPI_ISL_16956386_A/Peregrine_Falcon/England/256456/2023
EPI_ISL_17514128_A/mute_swan/Austria/23002053/2023
EPI_ISL_9616271_A/red_fox/Netherlands/22002190−003/2022
EPI_ISL_7996369_A/mute_swan/Netherlands/21039291−002/2021
EPI_ISL_11007532_A/swan/Slovenia/2072_22VIR777−5/2021
EPI_ISL_8650947_A/red_fox/Netherlands/22000097−004/2022
EPI_ISL_6675755_A/goose/Netherlands/21038413−002/2021
EPI_ISL_8799320_A/polecat/Netherlands/22000516−003/2022
EPI_ISL_15857990_A/Chicken/Italy/22VIR9832−3/2022
EPI_ISL_14864640_A/fox/England/M0025−12−21/2021
EPI_ISL_13486869_A/Greylag_goose/Scotland/024915/2022
EPI_ISL_13370511_A/turkey/Wales/065047/2021
EPI_ISL_15957774_A/Chicken/England/141990/2022
EPI_ISL_17267157_A/European_herring_gull/Netherlands/9/2023
EPI_ISL_17465832_A/harbour_porpoise/England/040497/2023
EPI_ISL_16811199_A/Mute_Swan/England/120693/2022
EPI_ISL_18594303_A_red_fox_Denmark_08303−1.01
EPI_ISL_17880911_A/Red_fox/Germany/51023−124/2023
EPI_ISL_7357590_A/mute_swan/Croatia/101/2021
EPI_ISL_11259298_A/turkey/Spain/489−21_22VIR2142−25/2022
EPI_ISL_11259310_A/turkey/Spain/645−1_22VIR2142−36/2022
EPI_ISL_7753387_A/Eurasian_wigeon/Germany−SH/AI06143/2021
EPI_ISL_9856775_A/chicken/Netherlands/21037287−006010/2021
EPI_ISL_13370419_A/chicken/England/064357/2021
EPI_ISL_14761496_A/chicken/Italy/22VIR1953−4/2022
EPI_ISL_14761129_A/turkey/Italy/21VIR11692/2021
EPI_ISL_9603937_A/pheasant/Czech_Republic/25827−2/2021
EPI_ISL_12325995_A/goose/Czech_Republic/25322−229/2021
EPI_ISL_19184045_A/chicken/Serbia/22337−21/2021
EPI_ISL_19029378_A/Fox/Bavaria/i193/2023
EPI_ISL_17971993_A/domestic_cat/Poland/H266−W/2023
EPI_ISL_17514173_A/black−headed_gull/Austria/23010977−001/2023
EPI_ISL_15857954_A/laying_hen/Italy/22VIR10267−7/2022
EPI_ISL_18612177_A/common_tern/Slovenia/PER942−23T2A_23VIR10323−6/2023
EPI_ISL_17679766_A/black−headed_gull/Italy/23VIR1203−4/2023
EPI_ISL_15878541_A/Mink/Spain/3691−2_22VIR10586−8/2022
EPI_ISL_17982393_A/Sandwich_Tern/Netherlands/7/2023
EPI_ISL_17821064_A/Black−headed_Gull/Netherlands/79/2023
EPI_ISL_18226743_A/red_fox/Northern_Ireland/22185/2023
EPI_ISL_17710034_A/Black−Headed_Gull/England/259311/2023
EPI_ISL_15957660_A/chicken/Wales/138294/2022
EPI_ISL_18255293_A/peregrine_falcon/Sweden/SVA230725SZ0382/FB002504/M−2023
EPI_ISL_16904555_A/Red_Fox/Wales/017836/2023
EPI_ISL_17710043_A/Black−Headed_Gull/England/125550/2023
EPI_ISL_18618701_A/mink/Finland/3076/2023
EPI_ISL_17880914_A/Red_fox/Germany/51023−261/2023
EPI_ISL_18432789_A/peregrine_falcon/Germany−NI/2023AI07594/2023
EPI_ISL_18112326_A/little_tern/England/093351/2023
EPI_ISL_17245141_A/Vulpes_vulpes/Belgium/01924_0003/2023
EPI_ISL_17245099_A/Vulpes_vulpes/Belgium/01784_0010/2023
EPI_ISL_17774358_A/Vulpes_Vulpes/Belgium/03704_0001/2023
EPI_ISL_18115436_A/black−headed_gull/Leningrad_region/RII−WD329S/2023
EPI_ISL_18033223_A/black−headed_gull/Poland/MB180−NMJ/2023
EPI_ISL_17233302_A/fox/France/23P002844/2023
EPI_ISL_17821062_A/Black−headed_Gull/Netherlands/77/2023
EPI_ISL_17716819_A/black−headed_gull/Czech_Republic/6947−4/2023
EPI_ISL_18728100_A/arctic−fox/Finland/677/2023
EPI_ISL_18122424_A/arctic−fox/Finland/544/2023
EPI_ISL_17699037_A/black−headed_gull_/Sweden/SVA230509SZ0614/FB001431/AB−2023
EPI_ISL_17716064_A/black−headed_gull/Austria/23030829−001/2023
EPI_ISL_13955204_A/chicken/Czech_Republic/8028−1/2022
0.03
group
Avian
HPAI in this study
Mammals

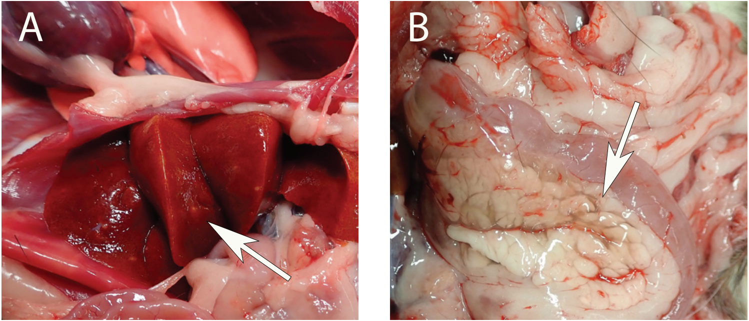


Supplementary Figure 2. Macroscopic changes after H5N1 infection: (A) liver (ferret group C) with multifocal light brown foci (arrow) consistent with necrosis; (B) pancreas (ferret group C), diffuse edema in pancreas (arrow) with multifocal necrosis (not visible in photo).

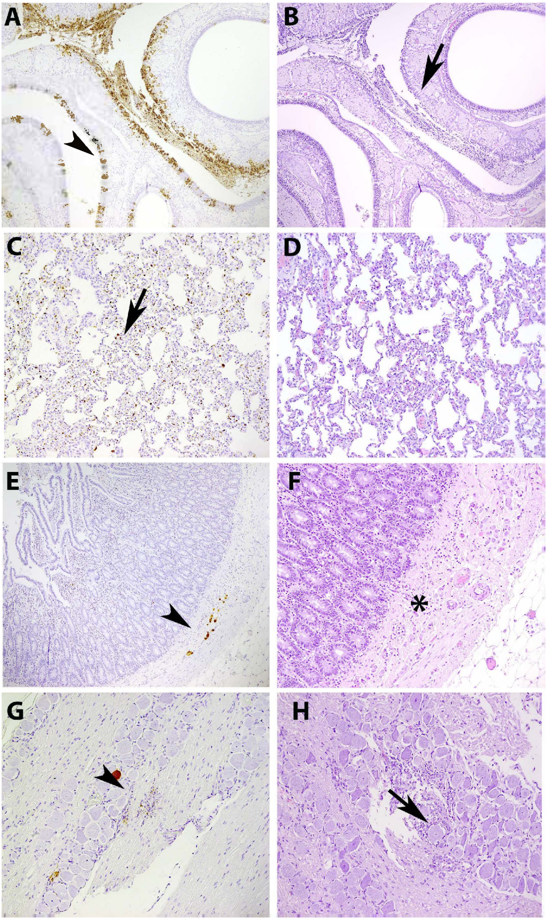


Supplementary Figure 3: H5N1 induced pathology and associated virus expression. (A-B) nasal conchae, moderate to extensive staining viral protein in olfactory epithelial cells (arrowhead) with degeneration, necrosis and loss of epithelial cells (arrow) ferret#23 group D, objective 10x; (C-D) lung, mild to moderate viral protein staining in individual mononuclear cells (arrowhead) with increased cellularity in alveolar walls and mild necrosis ferret#20 group C, objective 20x; (E-F) duodenum, multifocal viral protein staining in neurons in lamina muscularis (arrow) with increase of inflammatory cells (asterisk), ferret#15 group C, objective 10x and 20x, respectively; (G-H) trigeminal ganglion, viral protein staining in neuron and cellular debris (arrowhead) with individual necrosis of neurons (arrow) ferret#15 group C objective 20x; A, C, E and G immunohistochemistry influenza A nucleoprotein (NP) corresponding with HE; B, D, F and H haematoxylin and eosin stain (HE).

Supplementary Table 1: GISAID accession numbers. We gratefully acknowledge the authors, originating and submitting laboratories of the sequences from GISAID's EpiFlu™ Database on which this research is based. The list is detailed below. All submitters of data may be contacted directly via www.gisaid.org

| Isolate-ID | Isolate name | Country | Collection date | Originating Lab | Submitting Lab | Authors |
|---|---|---|---|---|---|---|
| EPI_ISL_10220427 | A/red fox/Netherlands/22002636-005/2022 | Netherlands | 2022-Feb-07 | Wageningen Bioveterinary Research | Wageningen Bioveterinary Research | Beerens, Nancy; Harders, Frank; Pritz-Verschuren, Sylvia; Roose, Marit; Venema, Sandra; Germeraad, Evelien; Engelsma, Marc; Heutink, Rene |
| EPI_ISL_10220429 | A/polecat/Netherlands/22002792-005/2022 | Netherlands | 2022-Feb-09 | Wageningen Bioveterinary Research | Wageningen Bioveterinary Research | Beerens, Nancy; Harders, Frank; Pritz-Verschuren, Sylvia; Roose, Marit; Venema, Sandra; Germeraad, Evelien; Engelsma, Marc; Heutink, Rene |
| EPI_ISL_10255581 | A/red fox/Netherlands/22002992-003/2022 | Netherlands | 2022-Feb-11 | Wageningen Bioveterinary Research | Wageningen Bioveterinary Research | Beerens, Nancy; Harders, Frank; Pritz-Verschuren, Sylvia; Roose, Marit; Venema, Sandra; Germeraad, Evelien; Engelsma, Marc; Heutink, Rene |
| EPI_ISL_10255587 | A/red fox/Netherlands/22002993-003 /2022 | Netherlands | 2022-Feb-11 | Wageningen Bioveterinary Research | Wageningen Bioveterinary Research | Beerens, Nancy; Harders, Frank; Pritz-Verschuren, Sylvia; Roose, Marit; Venema, Sandra; Germeraad, Evelien; Engelsma, Marc; Heutink, Rene |
| EPI_ISL_11007532 | A/swan/Slovenia/2072_22VIR777-5/2021 | Slovenia | 2021-Dec-30 | University of Ljubljana | Istituto Zooprofilattico Sperimentale delle Venezie | Slavec, B.; Ra?nik, J.; Krape?, U.; ?labravec, Z.; A?ko, J.; Cociancich, V.; Paller, T.; Vidrih, ?.; Rojs, O.Z.; Arseniev, S.; Groza, O.; Barbierato, G.; Zecchin, B.; Fusaro, A.; Schivo, A.; Salviato, A.; Palumbo, E.; Giussani, E.; Monne, I.; Terregino, C. |
| EPI_ISL_1122425 | A/chicken/England/043315/2020 | United Kingdo | 2020-Dec-15 | Animal and Plant Health Agency (APHA) | Animal and Plant Health Agency (APHA) | |
| EPI_ISL_11259283 | A/fox/Ireland/3866_22VIR2064-1/2022 | Ireland | 2022-Feb-14 | Central Veterinary Research Laboratory | Istituto Zooprofilattico Sperimentale delle Venezie | Byrne, C.; Garcia, K.; Cuartero, L.G.; Barbierato, G.; Zecchin, B.; Fusaro, A.; Schivo, A.; Salviato, A.; Palumbo, E.; Giussani, E.; Monne, I.; Terregino, C. |
| EPI_ISL_11259298 | A/turkey/Spain/489-21_22VIR2142-25/2022 | Spain | 2022-Feb-08 | Laboratorio Central de Veterinaria | Istituto Zooprofilattico Sperimentale delle Venezie | Ruano, M.J.; Rocha, A.; Sanchez, A.; Aguero, M; Barbierato, G.; Zecchin, B.; Fusaro, A.; Schivo, A.; Salviato, A.; Palumbo, E.; Giussani, E.; Monne, I.; Terregino, C. |
| EPI_ISL_11259310 | A/turkey/Spain/645-1_22VIR2142-36/2022 | Spain | 2022-Feb-15 | Laboratorio Central de Veterinaria | Istituto Zooprofilattico Sperimentale delle Venezie | Ruano, M.J.; Rocha, A.; Sanchez, A.; Aguero, M; Barbierato, G.; Zecchin, B.; Fusaro, A.; Schivo, A.; Salviato, A.; Palumbo, E.; Giussani, E.; Monne, I.; Terregino, C. |
| EPI_ISL_1139101 | A/swan/Netherlands/20017605-002/2020 | Netherlands | 2020-Nov-09 | Wageningen Bioveterinary Research | Wageningen Bioveterinary Research | Beerens, Nancy; Harders, Frank; Pritz-Verschuren, Sylvia; Roose, Marit; Germeraad, Evelien; Engelsma, Marc; Bossers, Alex; Heutink, Rene |
| EPI_ISL_1139102 | A/swan/Netherlands/20017772-002/2020 | Netherlands | 2020-Nov-10 | Wageningen Bioveterinary Research | Wageningen Bioveterinary Research | Beerens, Nancy; Harders, Frank; Pritz-Verschuren, Sylvia; Roose, Marit; Germeraad, Evelien; Engelsma, Marc; Bossers, Alex; Heutink, Rene |
| EPI_ISL_11406401 | A/chicken/England/000187/2022 | United Kingdo | 2022-Jan-03 | Animal and Plant Health Agency (APHA) | Animal and Plant Health Agency (APHA) | |
| EPI_ISL_11798575 | A/European_herring_gull/Denmark/19968-1.02/2021-05-14 | Denmark | 2021-May-14 | Statens Serumm Institute | Statens Serum Institute | Charlotte Hjulsager, Yuan Liang |
| EPI_ISL_12325995 | A/goose/Czech Republic/25322-229/2021 | Czech Repub | 2021-Dec-18 | State Veterinary Institute Prague | State Veterinary Institute Prague | Alexander,Nagy;Martina,Stara;Lenka,Cernikova |
| EPI_ISL_13370419 | A/chicken/England/064357/2021 | United Kingdo | 2021-Dec-01 | Animal and Plant Health Agency (APHA) | Animal and Plant Health Agency (APHA) | |
| EPI_ISL_13370511 | A/turkey/Wales/065047/2021 | United Kingdo | 2021-Dec-02 | Animal and Plant Health Agency (APHA) | Animal and Plant Health Agency (APHA) | |
| EPI_ISL_13486700 | A/Barnacle_goose/Scotland/003579/2022 | United Kingdo | 2022-Jan-02 | Animal and Plant Health Agency (APHA) | Animal and Plant Health Agency (APHA) | |
| EPI_ISL_13486869 | A/Greylag_goose/Scotland/024915/2022 | United Kingdo | 2022-Mar-07 | Animal and Plant Health Agency (APHA) | Animal and Plant Health Agency (APHA) | |
| EPI_ISL_13955204 | A/chicken/Czech Republic/8028-1/2022 | Czech Repub | 2022-Apr-13 | State Veterinary Institute Prague | State Veterinary Institute Prague | Alexander,Nagy;Lenka,Cernikova;Martina,Stara |
| EPI_ISL_14571432 | A/common tern/Germany-NI/AI03186/2022 | Germany | 2022-Jun-07 | Lebensmittel- und Veterinärinstitut Oldenburg - Standort Veterinärinstitut | Friedrich-Loeffler-Institut | |
| EPI_ISL_14760707 | A/chicken/Italy/21VIR10969-3/2021 | Italy | 2021-Dec-09 | Istituto Zooprofilattico Sperimentale delle Venezie, EU/OIE/Reference Laboratory and FAO Reference Centre for AI and ND | Istituto Zooprofilattico Sperimentale delle Venezie | Barbierato, G.; Zecchin, B.; Fusaro, A.; Schivo, A.; Salviato, A.; Palumbo, E.; Giussani, E.; Pastori, A.; Monne, I.; Terregino, C. |
| EPI_ISL_14761129 | A/turkey/Italy/21VIR11692/2021 | Italy | 2021-Dec-23 | Istituto Zooprofilattico Sperimentale delle Venezie, EU/OIE/Reference Laboratory and FAO Reference Centre for AI and ND | Istituto Zooprofilattico Sperimentale delle Venezie | Barbierato, G.; Zecchin, B.; Fusaro, A.; Schivo, A.; Salviato, A.; Palumbo, E.; Giussani, E.; Pastori, A.; Monne, I.; Terregino, C. |
| EPI_ISL_14761496 | A/chicken/Italy/22VIR1953-4/2022 | Italy | 2022-Feb-22 | Istituto Zooprofilattico Sperimentale delle Venezie, EU/OIE/Reference Laboratory and FAO Reference Centre for AI and ND | Istituto Zooprofilattico Sperimentale delle Venezie | Barbierato, G.; Zecchin, B.; Fusaro, A.; Schivo, A.; Salviato, A.; Palumbo, E.; Giussani, E.; Pastori, A.; Monne, I.; Terregino, C. |

| | | | | | | |
|---|---|---|---|---|---|---|
| EPI_ISL_14864640 | A/fox/England/M0025-12-21/2021 | United Kingdo | 2021-Dec-07 | Animal and Plant Health Agency (APHA) | Animal and Plant Health Agency (APHA) | |
| EPI_ISL_14864642 | A/otter/Scotland/M100366/2022 | United Kingdo | 2022-Jan-01 | Animal and Plant Health Agency (APHA) | Animal and Plant Health Agency (APHA) | |
| EPI_ISL_15267018 | A/Greylag Goose/Netherlands/14/2022 | Netherlands | 2022-Aug-31 | Erasmus Medical Center | Erasmus Medical Center | |
| EPI_ISL_15518269 | A/lynx/Finland/Vi209-22_22VIR3127-2/2022 | Finland | 2022-Feb-01 | Finnish Food Authority | Istituto Zooprofilattico Sperimentale Delle Venezie | Tammiranta, N.;Kantala, T.;Laamanen, I.;Gadd, T.;Zecchin, B.;Fusaro, A.;Schivo, A.;Salviato, A.;Palumbo, E.;Milani, A.;Giussani, E.;Pastori, A.;Monne, I.;Terregino, C. |
| EPI_ISL_15857954 | A/laying_hen/Italy/22VIR10267-7/2022 | Italy | 2022-Oct-25 | Istituto Zooprofilattico Sperimentale delle Venezie, EU/OIE/Reference Laboratory and FAO Reference Centre for AI and ND | Istituto Zooprofilattico Sperimentale delle Venezie | Barbierato, G.; Zecchin, B.; Fusaro, A.; Schivo, A.; Salviato, A.; Palumbo, E.; Giussani, E.; Pastori, A.; Monne, I.; Terregino, C. |
| EPI_ISL_15857990 | A/Chicken/Italy/22VIR9832-3/2022 | Italy | 2022-Oct-17 | Istituto Zooprofilattico Sperimentale delle Venezie, EU/OIE/Reference Laboratory and FAO Reference Centre for AI and ND | Istituto Zooprofilattico Sperimentale delle Venezie | Barbierato, G.; Zecchin, B.; Fusaro, A.; Schivo, A.; Salviato, A.; Palumbo, E.; Giussani, E.; Pastori, A.; Monne, I.; Terregino, C. |
| EPI_ISL_15878541 | A/Mink/Spain/3691-2_22VIR10586-8/2022 | Spain | 2022-Oct-18 | Laboratorio Central de Veterinaria | Istituto Zooprofilattico Sperimentale delle Venezie | Ruano, M.J.; Rocha, A.; Sanchez, A.; Aguero, M; Barbierato, G.; Zecchin, B.; Fusaro, A.; Schivo, A.; Salviato, A.; Palumbo, E.; Giussani, E.; Pastori, A.; Monne, I.; Terregino, C. |
| EPI_ISL_15925875 | A/Eurasian Wigeon/Netherlands/1/2022 | Netherlands | 2022-Oct-29 | Erasmus Medical Center | Erasmus Medical Center | |
| EPI_ISL_15957660 | A/chicken/Wales/138294/2022 | United Kingdo | 2022-Oct-21 | Animal and Plant Health Agency (APHA) | Animal and Plant Health Agency (APHA) | Byrne, A.; Howton, N.; Maskell, D.; Mollett, B.; Peers-Dent, J. |
| EPI_ISL_15957774 | A/Chicken/England/141990/2022 | United Kingdo | 2022-Oct-29 | Animal and Plant Health Agency (APHA) | Animal and Plant Health Agency (APHA) | Byrne, A.; Howton, N.; Maskell, D.; Mollett, B.; Peers-Dent, J. |
| EPI_ISL_15957953 | A/chicken/England/149805/2022 | United Kingdo | 2022-Nov-09 | Animal and Plant Health Agency (APHA) | Animal and Plant Health Agency (APHA) | Byrne, A.; Howton, N.; Maskell, D.; Mollett, B.; Peers-Dent, J. |
| EPI_ISL_16384167 | A/greylag_goose/England/316980/2022 | United Kingdo | 2022-Dec-04 | Animal and Plant Health Agency (APHA) | Animal and Plant Health Agency (APHA) | |
| EPI_ISL_16384182 | A/greylag_goose/England/119927/2022 | United Kingdo | 2022-Dec-06 | Animal and Plant Health Agency (APHA) | Animal and Plant Health Agency (APHA) | |
| EPI_ISL_16524596 | A/northern_gannet/Spain/22VIR12774-4_3104-40/2022 | Spain | 2022-Aug-30 | Istituto Zooprofilattico Sperimentale delle Venezie, EU/OIE/Reference Laboratory and FAO Reference Centre for AI and ND | Istituto Zooprofilattico Sperimentale Delle Venezie | Ruano,M.J.; Rocha,A.; Sanchez,A.; Aguero,M.; Pastori, A.; Zecchin, B.; Fusaro, A.; Schivo, A.; Salviato, A.; Palumbo, E.; Giussani, E.; Monne, I.; Terregino, C. |
| EPI_ISL_16549380 | A/Vulpes_vulpes/Belgium/14775-0001/2022 | Belgium | 2022-Dec-08 | Sciensano - Animal Infectious Diseases | Sciensano, Department of Animal Infectious Diseases | Van Borm, Steven; Roupie, Virginie; Hostyn, Pierre; Lambrecht, Benedicte; Steensels, Mieke |
| EPI_ISL_16811199 | A/Mute_Swan/England/120693/2022 | United Kingdo | 2022-Dec-30 | Animal and Plant Health Agency (APHA) | Animal and Plant Health Agency (APHA) | |
| EPI_ISL_16904555 | A/Red_Fox/Wales/017836/2023 | United Kingdo | 2023-Jan-12 | Animal and Plant Health Agency (APHA) | Animal and Plant Health Agency (APHA) | |
| EPI_ISL_16956386 | A/Peregrine_Falcon/England/256456/2023 | United Kingdo | 2023-Jan-07 | Animal and Plant Health Agency (APHA) | Animal and Plant Health Agency (APHA) | |
| EPI_ISL_17212547 | A/mute swan/England/SA14-234255/2020 | United Kingdo | 2020-Dec-03 | Animal and Plant Health Agency (APHA) | Animal and Plant Health Agency (APHA) | Seekings, AH |
| EPI_ISL_17233302 | A/fox/France/23P002844/2023 | France | 2023-Feb-10 | Anses (Ploufragan-Plouzané) | ANSES Agence Nationale De Securite Sanitaire De L'alimentation | |
| EPI_ISL_17245099 | A/Vulpes_vulpes/Belgium/01784_0010/2023 | Belgium | 2023-Feb-27 | Sciensano - Animal Infectious Diseases | Sciensano, Department of Animal Infectious Diseases | Van Borm, Steven; Roupie, Virginie; Hostyn, Pierre; Mathijs, Elisabeth; Lambrecht, Benedicte; Steensels, Mieke |
| EPI_ISL_17245141 | A/Vulpes_vulpes/Belgium/01924_0003/2023 | Belgium | 2023-Mar-02 | Sciensano - Animal Infectious Diseases | Sciensano, Department of Animal Infectious Diseases | Van Borm, Steven; Roupie, Virginie; Hostyn, Pierre; Mathijs, Elisabeth; Lambrecht, Benedicte; Steensels, Mieke |
| EPI_ISL_17267157 | A/European herring gull/Netherlands/9/2023 | Netherlands | 2023-Mar-03 | Erasmus Medical Center | Erasmus Medical Center | |
| EPI_ISL_17268132 | A/chicken/England/152082/2022 | United Kingdo | 2022-Nov-14 | Animal and Plant Health Agency (APHA) | Animal and Plant Health Agency (APHA) | |
| EPI_ISL_17447413 | A/red fox/Denmark/08658-1.01/2022 | Denmark | 2022-Apr-28 | Statens Serumm Institute | Statens Serum Institute | Hjulsager,Charlotte; Liang,Yuan |
| EPI_ISL_17465832 | A/harbour_porpoise/England/040497/2023 | United Kingdo | 2023-Feb-06 | Animal and Plant Health Agency (APHA) | Animal and Plant Health Agency (APHA) | |
| EPI_ISL_17514128 | A/mute_swan/Austria/23002053/2023 | Austria | 2023-Jan-10 | Institute for Veterinary Disease Control Moedling, Austrian Agency for Health and Food Safety | Austrian Agency for Health and Food Safety (AGES) | |
| EPI_ISL_17514173 | A/black-headed gull/Austria/23010977-001/2023 | Austria | 2023-Jan-30 | Institute for Veterinary Disease Control Moedling, Austrian Agency for Health and Food Safety | Austrian Agency for Health and Food Safety (AGES) | |
| EPI_ISL_17679766 | A/black-headed_gull/Italy/23VIR1203-4/2023 | Italy | 2023-Feb-10 | Istituto Zooprofilattico Sperimentale delle Venezie, EU/OIE/Reference Laboratory and FAO Reference Centre for AI and ND | Istituto Zooprofilattico Sperimentale Delle Venezie | Pastori, A.; Zecchin, B.; Fusaro, A.; Schivo, A.; Salviato, A.; Palumbo, E.; Giussani, E.; Monne, I.; Terregino, C. |
| EPI_ISL_17699037 | A/black-headed gull /Sweden/SVA230509SZ0614/FB001431/AB-2023 | Sweden | 2023-May-07 | Swedish Veterinary Agency (SVA) | Swedish Veterinary Agency (SVA) | "Siamak,Zohari" |
| EPI_ISL_17710034 | A/Black-Headed_Gull/England/259311/2023 | United Kingdo | 2023-Apr-14 | Animal and Plant Health Agency (APHA) | Animal and Plant Health Agency (APHA) | |
| EPI_ISL_17710043 | A/Black-Headed_Gull/England/125550/2023 | United Kingdo | 2023-Apr-28 | Animal and Plant Health Agency (APHA) | Animal and Plant Health Agency (APHA) | |
| EPI_ISL_17716064 | A/black-headed gull/Austria/23030829-001/2023 | Austria | 2023-Mar-08 | Austrian Agency for Health and Food Safety (AGES) | Austrian Agency for Health and Food Safety (AGES) | |
| EPI_ISL_17716819 | A/black-headed_gull/Czech_Republic/6947-4/2023 | Czech Repub | 2023-May-03 | State Veterinary Institute Prague | State Veterinary Institute Prague | Alexander,Nagy;Lenka,Cernikova;Martina,Stara |

| | | | | | | |
|---|---|---|---|---|---|---|
| EPI_ISL_17716831 | A/chicken/Czech_Republic/7531-3/2023 | Czech Repub | 2023-May-12 | State Veterinary Institute Prague | State Veterinary Institute Prague | Alexander,Nagy;Lenka,Cernikova;Martina,Stara |
| EPI_ISL_17774358 | A/Vulpes_Vulpes/Belgium/03704_0001/2023 | Belgium | 2023-Apr-14 | Sciensano - Animal Infectious Diseases | Sciensano, Department of Animal Infectious Diseases | Van Borm, Steven; Roupie, Virginie; Hostyn, Pierre; Mathijs, Elisabeth; Lambrecht, Benedicte; Steensels, Mieke |
| EPI_ISL_17791407 | A/Chicken/Netherlands/20019879-001005/2020 | Netherlands | 2020-Dec-14 | Wageningen Bioveterinary Research | Wageningen Bioveterinary Research | Beerens, Nancy; Harders, Frank; Pritz-Verschuren, Sylvia; Roose, Marit; Venema, Sandra; Germeraad, Evelien; Engelsma, Marc; Heutink, Rene |
| EPI_ISL_17821062 | A/Black-headed Gull/Netherlands/77/2023 | Netherlands | 2023-Feb-11 | Erasmus Medical Center | Erasmus Medical Center | |
| EPI_ISL_17821064 | A/Black-headed Gull/Netherlands/79/2023 | Netherlands | 2023-May-24 | Erasmus Medical Center | Erasmus Medical Center | |
| EPI_ISL_17880911 | A/Red fox/Germany/51023-124/2023 | Germany | 2023-Feb-09 | Lower Saxony State Office for Consumer Protection and Food Safety | Lower Saxony State Office for Consumer Protection & Food Safety | |
| EPI_ISL_17880914 | A/Red fox/Germany/51023-261/2023 | Germany | 2023-Mar-29 | Lower Saxony State Office for Consumer Protection and Food Safety | Lower Saxony State Office for Consumer Protection & Food Safety | |
| EPI_ISL_17971993 | A/domestic_cat/Poland/H266-W/2023(H5N1) | Poland | 2023-Jun-19 | National Veterinary Research Institut Poland, PIWet-PIB | National Veterinary Research Institut Poland, PIWet-PIB | Swieton, E.;Domanska-Blicharz, K.;Tarasiuk, K.; Wyrostek, K.;Stys-Fijol, N. |
| EPI_ISL_17982393 | A/Sandwich Tern/Netherlands/7/2023 | Netherlands | 2023-Jun-16 | Erasmus Medical Center | Erasmus Medical Center | |
| EPI_ISL_18006931 | A/buzzard/Germany-BB/AI00489/2022 | Germany | 2021-Dec-28 | Friedrich-Loeffler-Institut | Friedrich-Loeffler-Institut | |
| EPI_ISL_18033223 | A/black-headed_gull/Poland/MB180-NMJ/2023 | Poland | 2023-May-09 | National Veterinary Research Institut Poland, PIWet-PIB | National Veterinary Research Institut Poland, PIWet-PIB | Swieton,Edyta;Tarasiuk,Karolina;Wyrostek,Krzysztof;Kozak,Edyta;Stys-Fijol,Natalia;Smietanka,Krzysztof |
| EPI_ISL_18112326 | A/little_tern/England/093351/2023 | United Kingdo | 2023-Jul-11 | Animal and Plant Health Agency (APHA) | Animal and Plant Health Agency (APHA) | |
| EPI_ISL_18115436 | A/black-headed gull/Leningrad_region/RII-WD329S/2023 | Russian Fede | 2023-Aug-02 | WHO National Influenza Centre Russian Federation | WHO National Influenza Centre Russian Federation | Andrey, Komissarov; Artem, Fadeev; Nikita, Yolshin; Anna, Ivanova; Maria, Pisareva; Alena, Zheltukhina; Daria, Danilenko; Dmitry, Lioznov |
| EPI_ISL_18122424 | A/arctic-fox/Finland/544/2023 | Finland | 2023-Jul-14 | Finnish Food Authority | Finnish Institute for Health and Welfare, THL | Tuija Kantala, Alice Fusaro, Tuija Gadd, Monica Gallo, Niina Ikonen, Lauri Kareinen, Ari Kauppinen, Niina Tammiranta, Erika Lindh |
| EPI_ISL_18226743 | A/red fox/Northern Ireland/22185/2023 | United Kingdo | 2023-Jul-26 | AFBI - Agri-Food & Bioscience Institute | Agri-Food and Biosciences Institute (AFBINI) | Lagan, P.; Lemon, K. |
| EPI_ISL_18245812 | A/wild_bird/Poland/MW542/2021 | Poland | 2021-Dec-20 | National Veterinary Research Institut Poland, PIWet-PIB | National Veterinary Research Institute | Swieton, E.; Dziadek, K.; Kozak, E.; Wyrostek, K.; Tarasiuk, K.; Stys Fijol, N.; Smietanka, K. |
| EPI_ISL_18255293 | A/peregrine falcon/Sweden/SVA230725SZ0382/FB002504/M-2023 | Sweden | 2023-Jun-06 | Swedish Veterinary Agency (SVA) | Swedish Veterinary Agency (SVA) | "Siamak,Zohari" |
| EPI_ISL_18432789 | A/peregrine falcon/Germany-NI/2023AI07594/2023 | Germany | 2023-Sep-07 | Friedrich-Loeffler-Institut | Friedrich-Loeffler-Institut | |
| EPI_ISL_18455203 | A/Red_fox/Norway/2022-07-1768-2T/2022 | Norway | 2022-Jul-26 | Norwegian Veterinary Institute | Norwegian Veterinary Institute | Britt Gjerset, Cathrine Arnason Bøe |
| EPI_ISL_18458075 | A/mute_swan/Austria/2990-1_22VIR582-2/2021 | Austria | 2021-Dec-14 | Austrian Agency for Health and Food Safety (AGES) | Istituto Zooprofilattico Sperimentale Delle Venezie | Revilla-Fern?ndez, S.;Zecchin, B.;Pastori, A.;Fusaro, A.;Schivo, A.;Salviato, A.;Palumbo, E.;Giussani, E.;Monne, I.;Terregino, C. |
| EPI_ISL_18594303 | A_red_fox_Denmark_08303-1.01_2023-04-29 | Denmark | 2023-Apr-29 | Statens Serumm Institute | Statens Serum Institute | Hjulsager,C;Liang,Y |
| EPI_ISL_18612177 | A/common_tern/Slovenia/PER942-23T2A_23VIR10323-6/2023 | Slovenia | 2023-May-16 | University of Ljubljana | Istituto Zooprofilattico Sperimentale Delle Venezie | Salomoni, A.; Slavec, B.; Cociancich, V.; Zecchin, B.; Fusaro, A.; Schivo, A.; Salviato, A.; Palumbo, E.; Giussani, E.; Pastori, A.; Savegnago, E.; Monne, I.; Terregino, C. |
| EPI_ISL_18618701 | A/mink/Finland/3076/2023 | Finland | 2023-Sep-20 | Finnish Food Authority | Finnish Food Authority | Kauppinen, Ari; Kareinen, Lauri; Tammiranta, Niina; Kantala, Tuija; Fusaro, Alice; Gadd, Tuija |
| EPI_ISL_18728100 | A/arctic-fox/Finland/677/2023 | Finland | 2023-Aug-01 | Finnish Food Authority | Finnish Food Authority | Kauppinen, Ari; Kareinen, Lauri; Tammiranta, Niina; Kantala, Tuija; Fusaro, Alice; Giussani, Edoardo; Gadd, Tuija |
| EPI_ISL_19028669 | A/Est/Pärnu/Seagull/TA21-13284-5/2021 | Estonia | 2021-Jun-02 | National Centre for Laboratory Research and Risk Assessment | The National Centre for Laboratory Research and Risk Assessment | Vilem, Annika; Juurik,Triinu; Nurmoja, Imbi |
| EPI_ISL_19029378 | A/Fox/Bavaria/i193/2023 | Germany | 2023-Jun-12 | Bayerisches Landesamt für Gesundheit und Lebensmittelsicherheit | Bayerisches Landesamt für Gesundheit und Lebensmittelsicherheit | |
| EPI_ISL_19057416 | A/red fox/Netherlands/21040099-006_PB2E/2021 | Netherlands | 2021-Dec-03 | Wageningen Bioveterinary Research | Wageningen Bioveterinary Research | Beerens, Nancy; Harders, Frank; Pritz-Verschuren, Sylvia; Roose, Marit; Venema, Sandra; Germeraad, Evelien; Engelsma, Marc; Heutink, Rene |
| EPI_ISL_19057417 | A/red fox/Netherlands/21040099-006_PB2K/2021 | Netherlands | 2021-Dec-03 | Wageningen Bioveterinary Research | Wageningen Bioveterinary Research | Beerens, Nancy; Harders, Frank; Pritz-Verschuren, Sylvia; Roose, Marit; Venema, Sandra; Germeraad, Evelien; Engelsma, Marc; Heutink, Rene |
| EPI_ISL_19184045 | A/chicken/Serbia/22337-21/2021 | Serbia | 2021-May-01 | Scientific Veterinary Institute Novi Sad | | Gajdov,V., Petrovic,T., Lazic,G., Djurdjevic,B., Pajic,M., Knezevic,S.; Nisavic,J. |

| | | | | | | |
|---|---|---|---|---|---|---|
| EPI_ISL_2194218 | A/red fox/Netherlands/21028774-002/2021 | Netherlands | 2021-May-17 | Wageningen Bioveterinary Research | Wageningen Bioveterinary Research | Beerens, Nancy; Harders, Frank; Pritz-Verschuren, Sylvia; Roose, Marit; Germeraad, Evelien; Engelsma, Marc; Bossers, Alex; Heutink, Rene |
| EPI_ISL_603133 | A/Eurasian Wigeon/Netherlands/1/2020 | Netherlands | 2020-Oct-16 | Erasmus Medical Center | Erasmus Medical Center | |
| EPI_ISL_603134 | A/Eurasian Wigeon/Netherlands/4/2020 | Netherlands | 2020-Oct-16 | Erasmus Medical Center | Erasmus Medical Center | |
| EPI_ISL_603135 | A/Eurasian Wigeon/Netherlands/5/2020 | Netherlands | 2020-Oct-16 | Erasmus Medical Center | Erasmus Medical Center | |
| EPI_ISL_632314 | A/greylag goose/Netherlands/20016582-004/2020 | Netherlands | 2020-Oct-28 | Wageningen Bioveterinary Research | Wageningen Bioveterinary Research | Beerens, Nancy; Harders, Frank; Verschuren-Pritz, Sylvia; Roose, Marit; Germeraad, Evelien; Engelsma, Marc; Bossers, Alex; Heutink, Rene |
| EPI_ISL_632315 | A/eurasian teal/Netherlands/20016896-013/2020 | Netherlands | 2020-Nov-02 | Wageningen Bioveterinary Research | Wageningen Bioveterinary Research | Beerens, Nancy; Harders, Frank; Verschuren-Pritz, Sylvia; Roose, Marit; Germeraad, Evelien; Engelsma, Marc; Bossers, Alex; Heutink, Rene |
| EPI_ISL_632316 | A/eurasian curlew/Netherlands/20016890-001/2020 | Netherlands | 2020-Nov-01 | Wageningen Bioveterinary Research | Wageningen Bioveterinary Research | Beerens, Nancy; Harders, Frank; Verschuren-Pritz, Sylvia; Roose, Marit; Germeraad, Evelien; Engelsma, Marc; Bossers, Alex; Heutink, Rene |
| EPI_ISL_6675755 | A/goose/Netherlands/21038413-002/2021 | Netherlands | 2021-Nov-08 | Wageningen Bioveterinary Research | Wageningen Bioveterinary Research | Beerens, Nancy; Harders, Frank; Pritz-Verschuren, Sylvia; Roose, Marit; Germeraad, Evelien; Engelsma, Marc; Heutink, Rene |
| EPI_ISL_6761000 | A/greylag goose/Netherlands/21037809-001/2021 | Netherlands | 2021-Oct-31 | Wageningen Bioveterinary Research | Wageningen Bioveterinary Research | Beerens, Nancy; Harders, Frank; Pritz-Verschuren, Sylvia; Roose, Marit; Germeraad, Evelien; Engelsma, Marc; Heutink, Rene |
| EPI_ISL_683592 | A/Eurasian_wigeon/Italy/20VIR7301-206/2020 | Italy | 2020-Nov-21 | Istituto Zooprofilattico Sperimentale delle Venezie, EU/OIE/Reference Laboratory and FAO Reference Centre for AI and ND | Istituto Zooprofilattico Sperimentale Delle Venezie | Zecchin, B.; Fusaro, A.; Pastori, A.; Milani, A.; Salviato, A.; Schivo, A.; Monne, I.; Terregino, C. |
| EPI_ISL_711055 | A/chicken/Netherlands/20019879-001005/2020 | Netherlands | 2020-Dec-14 | Wageningen Bioveterinary Research | Wageningen Bioveterinary Research | Beerens, Nancy; Harders, Frank; Pritz-Verschuren, Sylvia; Roose, Marit; Germeraad, Evelien; Engelsma, Marc; Bossers, Alex; Heutink, Rene |
| EPI_ISL_711059 | A/greylag goose/Netherlands/20019685-002/2020 | Netherlands | 2020-Dec-08 | Wageningen Bioveterinary Research | Wageningen Bioveterinary Research | Beerens, Nancy; Harders, Frank; Pritz-Verschuren, Sylvia; Roose, Marit; Germeraad, Evelien; Engelsma, Marc; Bossers, Alex; Heutink, Rene |
| EPI_ISL_7357590 | A/mute swan/Croatia/101/2021 | Croatia | 2021-Nov-12 | Croatian Veterinary Institute, Poultry Centre | Croatian Veterinary Institute | Savić, Vladimir |
| EPI_ISL_766876 | A/mute_swan/England/234255/2020 | United Kingdo | 2020-Dec-03 | Animal and Plant Health Agency (APHA) | Animal and Plant Health Agency (APHA) | |
| EPI_ISL_7683101 | A/common buzzard/Netherlands/21039607-002/2021 | Netherlands | 2021-Nov-25 | Wageningen Bioveterinary Research | Wageningen Bioveterinary Research | Beerens, Nancy; Harders, Frank; Pritz-Verschuren, Sylvia; Roose, Marit; Venema, Sandra; Germeraad, Evelien; Engelsma, Marc; Heutink, Rene |
| EPI_ISL_7753387 | A/Eurasian wigeon/Germany-SH/AI06143/2021 | Germany | 2021-Oct-22 | Landeslabor Schleswig-Holstein | Friedrich-Loeffler-Institut | |
| EPI_ISL_7952119 | A/great black-backed gull/Netherlands/21039609-001-002/2021 | Netherlands | 2021-Nov-28 | Wageningen Bioveterinary Research | Wageningen Bioveterinary Research | Beerens, Nancy; Harders, Frank; Pritz-Verschuren, Sylvia; Roose, Marit; Venema, Sandra; Germeraad, Evelien; Engelsma, Marc; Heutink, Rene |
| EPI_ISL_7952121 | A/sanderling/Netherlands/21039528-002/2021 | Netherlands | 2021-Nov-22 | Wageningen Bioveterinary Research | Wageningen Bioveterinary Research | Beerens, Nancy; Harders, Frank; Pritz-Verschuren, Sylvia; Roose, Marit; Venema, Sandra; Germeraad, Evelien; Engelsma, Marc; Heutink, Rene |
| EPI_ISL_7996369 | A/mute swan/Netherlands/21039291-002/2021 | Netherlands | 2021-Nov-19 | Wageningen Bioveterinary Research | Wageningen Bioveterinary Research | Beerens, Nancy; Harders, Frank; Pritz-Verschuren, Sylvia; Roose, Marit; Venema, Sandra; Germeraad, Evelien; Engelsma, Marc; Heutink, Rene |
| EPI_ISL_8650947 | A/red fox/Netherlands/22000097-004/2022 | Netherlands | 2022-Jan-03 | Wageningen Bioveterinary Research | Wageningen Bioveterinary Research | Beerens, Nancy; Harders, Frank; Pritz-Verschuren, Sylvia; Roose, Marit; Venema, Sandra; Germeraad, Evelien; Engelsma, Marc; Heutink, Rene |
| EPI_ISL_8799320 | A/polecat/Netherlands/22000516-003/2022 | Netherlands | 2022-Jan-08 | Wageningen Bioveterinary Research | Wageningen Bioveterinary Research | Beerens, Nancy; Harders, Frank; Pritz-Verschuren, Sylvia; Roose, Marit; Venema, Sandra; Germeraad, Evelien; Engelsma, Marc; Heutink, Rene |
| EPI_ISL_956412 | A/greater_white-fronted_goose/Italy/20VIR8073-4/2020 | Italy | 2020-Nov-23 | Istituto Zooprofilattico Sperimentale delle Venezie, EU/OIE/Reference Laboratory and FAO Reference Centre for AI and ND | Istituto Zooprofilattico Sperimentale Delle Venezie | Zecchin, B.; Fusaro, A.; Milani, A.; Schivo, A.; Salviato, A.; Pastori, A.; Zamperin, G.; Monne, I.; Terregino, C. |
| EPI_ISL_9603937 | A/pheasant/Czech Republic/25827-2/2021 | Czech Repub | 2021-Dec-25 | State Veterinary Institute Prague | State Veterinary Institute Prague | Alexander,Nagy;Lenka,Cernikova;Martina,Stara |

| | | | | | | |
|---|---|---|---|---|---|---|
| EPI_ISL_9616271 | A/red fox/Netherlands/22002190-003/2022 | Netherlands | 2022-Jan-31 | Wageningen Bioveterinary Research | Wageningen Bioveterinary Research | Beerens, Nancy; Harders, Frank; Pritz-Verschuren, Sylvia; Roose, Marit; Venema, Sandra; Germeraad, Evelien; Engelsma, Marc; Heutink, Rene |
| EPI_ISL_9856775 | A/chicken/Netherlands/21037287-006010/2021 | Netherlands | 2021-Oct-25 | Wageningen Bioveterinary Research | Wageningen Bioveterinary Research | Beerens, Nancy; Harders, Frank; Pritz-Verschuren, Sylvia; Roose, Marit; Venema, Sandra; Germeraad, Evelien; Engelsma, Marc; Heutink, Rene; Luca, Bordes |

Supplementary Table 2: Detailed scoring clinical signs

| | Depression |
|---|---|
| 0 | Animal is active, bright, alert and responds quickly to the environment or handling |
| 1 | Animal is less active (sits hunched over), but reacts normally |
| 2 | Animal is inactive and becomes less reactive, is clearly lethargic, withdraws from the group |
| 3 | Animal is very lethargic and absent-minded and shows no response during handling |

| | Nasal discharge |
|---|---|
| 0 | Absent |
| 1 | Slight and intermittent watery to mucous discharge (teary eye, wet nose) |
| 2 | Moderate and persistent watery, mucopurulent discharge (tear streak eye, runny nose) |
| 3 | Profuse watery eye discharge and or bloody nasal discharge |

| | Breathing |
|---|---|
| 0 | Normal breathing frequency |
| 1 | Slightly increased respiratory rate and/or respiratory sounds (rattling, wheezing) |
| 2 | Clearly increased respiratory rate, clear abdominal respiratory sounds (gurgling, rasping, shortness of breath) |
| 3 | Very high respiratory rate or severe abdominal breathing (open mouth, shortness of breath) |

| | Sneezing/ coughing |
|---|---|
| 0 | Absent |
| 1 | Occasional sneezing/coughing (only after handling or during activity) |
| 2 | Frequent sneezing/coughing (even at rest) |
| 3 | Frequent sneezing/coughing at rest with prolonged episodes (attack) |

| | Neurological signs |
|---|---|
| 0 | Absent |
| 1 | Mild ataxia, muscle tremors, jerky movements, less coordinated movements |
| 2 | Marked ataxia (impaired balance/disorientation, weakness in the hindquarters) |
| 3 | Severe ataxia (uncontrolled movements, twisted neck, complete paralysis of the hindquarters) |

| | Diarrhea |
|---|---|
| 0 | Normal stools, no abnormalities |
| 1 | Faeces of abnormal color or consistency |
| 2 | Faeces of abnormal color and consistency |
| 3 | Faeces with abnormal color and consistency and clearly increased in quantity (possibly with blood admixture) |

Supplementary Table 2. Weight loss, relative lung weight, clinical signs and gross pathology changes for each animal.

| Group | Animal number | Scheduled necropsy DPI | Exit/necropsy DPI | Weight arrival (gr) | Weight 0 DPI (gr) | Weight necropsy (gr) | Lung weight (gr) | Weight loss arrival vs 0 DPI (%) | Weight loss 0 DPI untill necropsy (%) | Lung body weight ratio at necropsy (%) | Clinical signs before necropsy | Macroscopically affected lungs | Lung consoli dation% | Hepatitis | Edema necrose pancreas |
|---|---|---|---|---|---|---|---|---|---|---|---|---|---|---|---|
| A | 2 | 4 | 4 | 1500 | 1520 | 1521 | 11.9 | -1 | 0 | 0.8 | 0 | | 0 | | |
| A | 3 | 4 | 4 | 1220 | 1268 | 1254 | 11.35 | -4 | 1 | 0.9 | 0 | | 0 | | |
| A | 4 | 4 | 4 | 1351 | 1396 | 1378 | 11.15 | -3 | 1 | 0.8 | 0 | | 0 | | |
| A | 5 | 14 | 14 | 1240 | 1287 | 1281 | 12.01 | -4 | 0 | 0.9 | 0 | | 0 | | |
| A | 6 | 14 | 14 | 1553 | 1660 | 1609 | 12.9 | -7 | 3 | 0.8 | 0 | | 0 | | |
| A | 7 | 14 | 14 | 1252 | 1273 | 1239 | 10.12 | -2 | 3 | 0.8 | 0 | | 0 | | |
| B | 10 | 4 | 4 | 1225 | 1262 | 1118 | 9.95 | -3 | 11 | 0.9 | respiratory (1), neurologic (1), depression (1) | | 0 | | |
| B | 11 | 4 | 4 (HEP) | 1330 | 1310 | 1229 | 10.44 | 2 | 6 | 0.8 | respiratory (2), neurologic (1), depression (1) | | 2 | x | |
| B | 12 | 4 | 4 (HEP) | 1411 | 1464 | 1299 | 11.35 | -4 | 11 | 0.9 | respiratory (2), neurologic (1), depression (1) | x | 5 | x | |
| B | 9 | 14 | 4 (HEP) | 1328 | 1418 | 1245 | 12.1 | -7 | 12 | 1.0 | neurologic (2), depression(2) | x | 2 | x | |
| B | 14 | 14 | 5 (HEP) | 1386 | 1380 | 1180 | 14.24 | 0 | 14 | 1.2 | neurologic (2), depression (2) | x | 2 | x | x |
| B | 15 | 14 | 5 (HEP) | 1219 | 1199 | 1060 | 11.8 | 2 | 12 | 1.1 | neurologic (2), depression (3) | x | 20 | | |
| C | 16 | 4 | 4 | 1289 | 1287 | 1144 | 10.45 | 0 | 11 | 0.9 | respiratory (1), depresssion (1) | x | 10 | x | x |
| C | 17 | 4 | 4 | 1429 | 1520 | 1332 | 15.76 | -6 | 12 | 1.2 | depression (1) | | 0 | x | x |
| C | 21 | 4 | 2 (HEP) | 1738 | 1450 | 1393 | 29.4 | 17 | 4 | 2.1 | respiratory (3) | x | 60 | | |
| C | 18 | 14 | 5 (HEP) | 1423 | 1470 | 1268 | 13.85 | -3 | 14 | 1.1 | neurologic (2), depression (2) | | 0 | x | x |
| C | 19 | 14 | 4 (HEP) | 1135 | 1143 | 978 | 8.77 | -1 | 14 | 0.9 | respiratory (2), sneezing, coughing (1), neurologic (1), depression (1) | | 5 | x | |
| C | 20 | 14 | 4 (HEP) | 1109 | 1199 | 1033 | 11.85 | -8 | 14 | 1.1 | fournd dead, neurologic (1), depression (1) | x | 50 | x | |
| D | 22 | 14 | 7 (HEP) | 1160 | 1172 | 1030 | 11.4 | -1 | 12 | 1.1 | neurologic (2), depression (2) | | 2 | x | x |
| D | 23 | 14 | 7 (HEP) | 1651 | 1644 | 1385 | 12.8 | 0 | 16 | 0.9 | respiratory (1), neurologic (3), depression (3) | x | 8 | x | x |
| D | 24 | 14 | 7 (HEP) | 1380 | 1355 | 1156 | 11.25 | 2 | 15 | 1.0 | neurologic (2), depression(3) | x | 2 | x | x |
| E | 1 | 0 | 0 | 1368 | 1405 | 1405 | 10.76 | -3 | 0 | 0.8 | 0 | | 0 | | |
| E | 8 | 0 | 0 | 1303 | 1370 | 1370 | 12.14 | -5 | 0 | 0.9 | 0 | | 0 | | |
| E | 13 | 0 | 0 | 1461 | 1211 | 1211 | na | 17 | 0 | na | 0 | | 0 | | |
